# Co-amplification of CBX3 with EGFR or RAC1 in human cancers corroborated by a conserved genetic interaction among the genes

**DOI:** 10.1038/s41420-023-01598-5

**Published:** 2023-08-26

**Authors:** Giuseppe Bosso, Francesca Cipressa, Liliana Tullo, Giovanni Cenci

**Affiliations:** 1 Department of Biology and Biotechnology “C. Darwin”, Sapienza Università di Roma, Rome, Italy; 2 Department of Ecological and Biological Sciences, Università degli Studi della Tuscia, Viterbo, Italy; 3 Fondazione Cenci Bolognetti, Istituto Pasteur Italia, Rome, Italy; 4 Present Address: Telomeres and Telomerase Group, Molecular Oncology Program, Spanish National Cancer Centre (CNIO), Melchor Fernández Almagro 3, Madrid, E-28029 Spain

**Keywords:** Cancer epigenetics, RNAi, Epistasis

## Abstract

Chromobox Protein 3 (CBX3) overexpression is a common event occurring in cancer, promotes cancer cell proliferation and represents a poor prognosis marker in a plethora of human cancers. Here we describe that a wide spectrum of human cancers harbors a co-amplification of CBX3 gene with either EGFR or RAC1, which yields a statistically significant increase of both mRNA and protein levels of CBX3, EGFR and RAC1. We also reveal that the simultaneous overexpression of CBX3, RAC1 and EGFR gene products correlates with a worse prognosis compared to the condition when CBX3, RAC1 and EGFR are singularly upregulated. Furthermore, we also show that a co-occurrence of low-grade amplification, in addition to high-grade amplification, between CBX3 and EGFR or RAC1 is associated with a reduced patient lifespan. Finally, we find that CBX3 and RAC1/EGFR genetically interact in the model organism *Drosophila melanogaster*, suggesting that the simultaneous overexpression as well as well the co-occurrence of high- or low-grade copy number alterations in these genes is not accidental and could reflect evolutionarily conserved functional relationships.

## Introduction

Chromobox protein homolog 3 / Heterochromatin protein γ (CBX3/HP1γ), a member of the evolutionarily-conserved heterochromatin protein family, is a well-known DNA-binding factor playing multiple roles in gene transcriptional regulation [1, 2]. A large number of evidence has been accumulated over the years about a pivotal role of CBX3 in tumorigenesis [3, 4]. Indeed, cells that overexpress/upregulate CBX3, elicit cancer proliferation properties [3, 5, 6] and their persistence is considered a poor prognosis marker in a plethora of human cancers such as glioblastoma multiforme (GBM) [7], non-small cell lung cancer (NSCLC) [8], ovarian cancer [9], breast cancer (BRCA) [10, 11], osteosarcoma [5], hepatocellular carcinoma [6], gastric cancer [12], pancreatic adenocarcinoma [4] and prostate cancer [13]. Very recently, gene amplification emerged as a potential mechanism that underlies CBX3 overexpression in human cancer [11, 14] and consequently as a poor prognosis marker in NSCLC [14].

It has been also hypothesized that the oncogenic variant of the epidermal growth factor receptor (EGFR), a well-known proto-oncogene widely overexpressed and/or amplified in human cancer [15, 16], may be correlated with CBX3 expression in NSCLC [8]. Moreover, CBX3 has been also shown to stabilize in an indirect fashion EGFR expression in GBM [17]. The Ras-related C3 botulinum toxin substrate 1 (RAC1), a small signaling GTPase, has been found to be indirectly regulated by CBX3 during tumor progression [18], and its gene has been recently found to be amplified in cancer [19, 20]. Albeit CBX3, EGFR and RAC1 genes map inside three vast regions of the short arm of chromosome 7, namely 7p15.2-14.1, 7p12.3-11.2 and 7p22.3-21.1, respectively, which are also frequently amplified in lung adenocarcinoma [21], very little is known about the genetic and functional interactions in vivo occurring among CBX3 and either EGFR or RAC1 proto-oncogene and how they contribute to cancer aggressiveness and patient survival. More importantly, how such functional interactions can be exploited by tumors during the evolutive process of cell transformation remains still largely unexplored.

Here, we examined copy number, mRNA expression as well as survival curve data from several tumors and revealed that a co-amplification of CBX3 gene with either EGFR or RAC1 gene is a molecular event occurring in a wide spectrum of human cancers and yields a statistically significant increase of both mRNA and protein levels of CBX3, EGFR and RAC1. In addition, we show a co-occurrence of CBX3 low-grade copy number alteration with those of either EGFR or RAC1 and hypothesize that these events underlie an unprecedented functional relationship among these three genes. Finally, leveraging the UAS/GAL4 induced-RNA interference in the model organism *Drosophila melanogaster*, we demonstrate for the first time that the fly ortholog of CBX3 genetically interacts with both EGFR and RAC1 orthologs. These data support our view that the co-amplification of these three genes that could facilitate cancer development and proliferation is not a casual event but is sustained by evolutionarily conserved genetic and molecular interactions.

## Results

### CBX3 gene amplification co-occurs with EGFR and RAC1 gene amplification in several human cancers

**Fig. 1:**
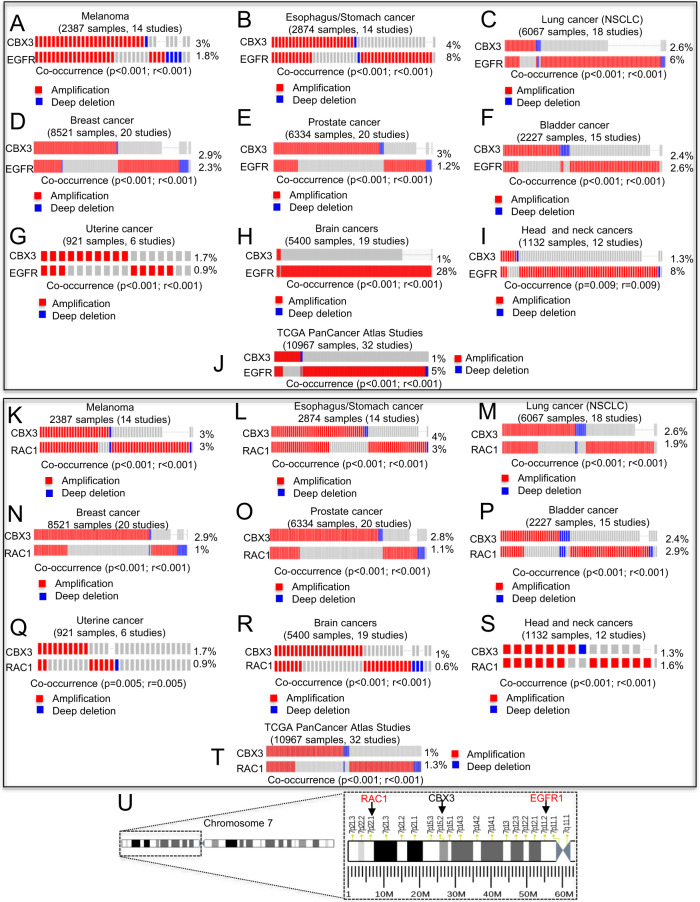
CBX3 locus amplification co-occurs with EGFR and RAC1 gene amplification in human cancers. **A**–**J** Oncoprints showing co-occurrence of amplification between CBX3 and EGFR genes in (**A**) melanoma, (**B**) Esophagus and stomach cancer, (**C**) Non-small cell lung cancer, (**D**) breast cancer, (**E**) prostate cancer, (**F**) bladder cancer, (**G**) uterine cancer, (**H**) brain cancer, (**I**) head and neck cancer, (**J**) TCGA PanCancer Atlas Studies. **K**–**T** Oncoprints showing co-occurrence of amplification between CBX3 and RAC1 genes in (**K**) melanoma, (**L**) esophagus and stomach cancer, (**M**) non-small cell lung cancer, (**N**) breast cancer, (**O**) prostate cancer, (**P**) bladder cancer, (**Q**) uterine cancer, (**R**) brain cancer, (**S**) head and neck cancer, (**T**) TCGA PanCancer Atlas Studies. The percentages on the right side of the oncoprints represent the frequency of gene amplification occurring in a given human cancer type. **U** Schematic representation of chromosome localization of *RAC1, CBX3 and EGFR* loci.

By querying cBioportal and TCGA databases, we selected data from some human cancers with the highest frequency of CBX3 gene amplification (Supplementary Fig. 1A). The analysis of the oncoprints of all selected cancer types revealed that CBX3 gene amplification strongly co-occurs with both EGFR and RAC1 gene amplification, thus suggesting that this event is not a cancer-specific phenomenon but encompasses diverse types of human cancers, regardless the nature of tissue/organ from which tumors arise (Fig. 1A–T).

The TCGA Pan Cancer Atlas specimens showing simultaneous amplification of CBX3 with either EGFR or RAC1 genes revealed no significant changes in either the aneuploidy score or the fraction of genome altered compared to diploid tumor samples (Supplementary Fig. 1B–E), thus, rendering unlikely that gene co-amplification may result from an increased genome instability. Moreover, the chromosome localization of the three genes on distant regions of the short arm of chromosome 7 (Fig. 1U) suggests that this co-amplification is more due to independent amplifications at different genic loci, rather than to a single amplification event on one single amplicon.

### Amplification of EGFR gene is associated to a transcriptional increase of CBX3 and vice versa

**Fig. 2:**
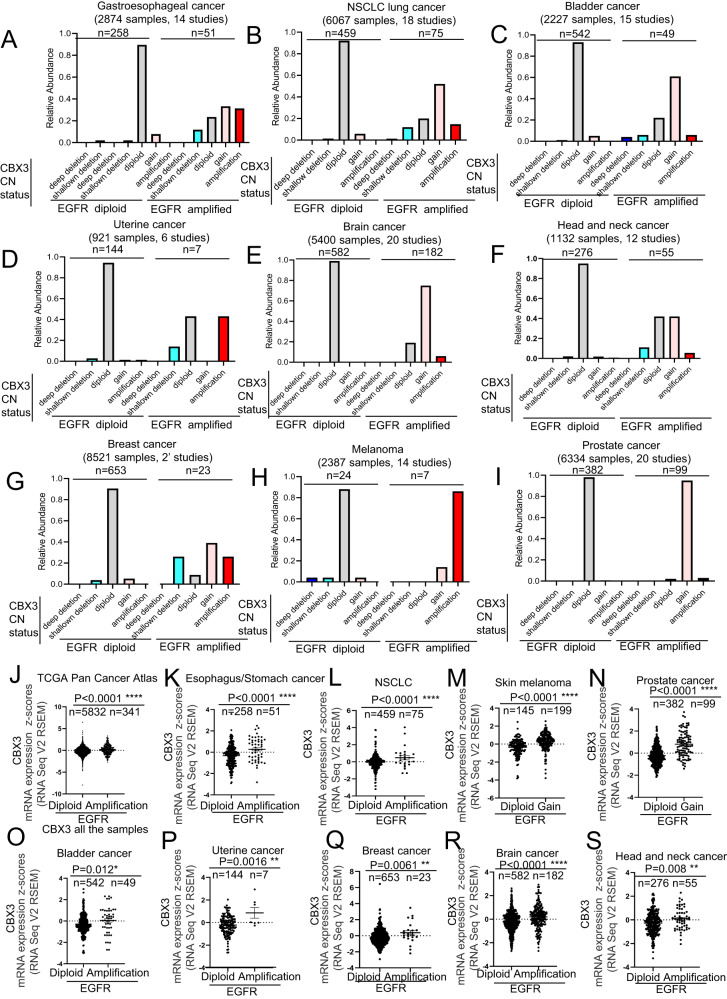
EGFR gene amplification is associated to increased levels of CBX3 transcripts. **A**–**I** Charts showing that EGFR gene amplification is coupled to a low-grade and high-grade increase in CBX3 gene copy number in (**A**) esophagus and stomach cancers, (**B**) non-small cell lung cancer, (**C**) urothelial cancer, (**D**) endometrial cancer, (**E**) brain cancer, (**F**) head and neck cancer, (**G**) breast cancer, (**H**) skin melanoma, (**I**) prostate cancer. **J**–**S** Charts showing that the samples harboring EGFR gene amplification display increased expression of CBX3 mRNA in the (**J**) TCGA Pan Cancer Atlas cohort, (**K**) esophagus and stomach cancers, (**L**) non-small cell lung cancer, (**M**) skin melanoma, (**N**) prostate cancer, (**O**) urothelial cancer, (**P**) uterine cancer, (**Q**) breast cancer, (**R**) brain cancer, (**S**) head and neck cancer. Data are expressed as mean ± SEM; *n* = samples per group. *P < 0.05; **P < 0.01; ***P < 0.001, ****P < 0.0001, ns not significant. T Student’s test unpaired (**J**–**S**).

We next studied more in detail the CBX3 copy number (CN) status in tumor samples (shown in Fig. 1) displaying either diploid or amplified EGFR gene. We found that, whereas in the 85–95% of the cases EGFR diploidy was associated with the CBX3 diploidy (Fig. 2A–I), the tumors displaying gene amplification of EGFR locus show a dramatic enrichment in the frequency of both low (gain; 10–90% of tumor specimens) and high (amplification; 5–80%) increase of CBX3 CN (Fig. 2A–I). Coherently, tumors with CBX3 gene amplification display a low gain increase or high-grade amplification of EGFR gene, thus confirming the evidence that CBX3 and EGFR gene amplification co-occurs in diverse human cancers (Supplementary Fig. 2A–I).

Next, we found that the presence of multiple copies in CBX3 and EGFR genes results in the upregulation of the corresponding mRNAs (Supplementary Figs. 3A–J and 4A–J). Interestingly, the analysis of the correlation between mRNA levels and the CN status also revealed that the cancer samples with amplified EGFR show a statistically significant increase of CBX3 mRNA expression regardless the tumor tissue of origin (Fig. 2J–S). On the other hand, cancer samples from TCGA Pan Cancer Atlas Studies harboring high CN increase of CBX3 display enhanced expression of EGFR at the transcriptional level (Supplementary Fig. 4K), further confirming the tight relationship between the CNVs occurring in CBX3 and EGFR genes in human cancer. Collectively, these data suggest that the co-occurrence of CBX3 and EGFR gene amplification is a frequent event in cancer that ultimately leads to an increased expression of both genes.

### Amplification of CBX3 gene is associated to transcriptional increase of RAC1 and vice versa

**Fig. 3:**
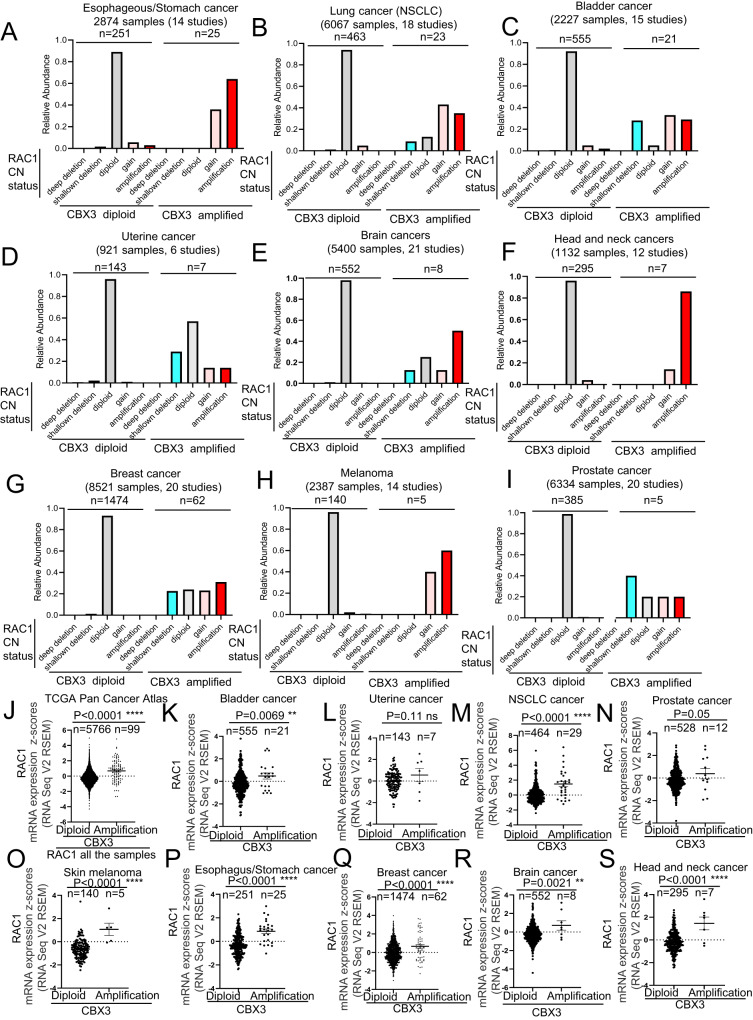
CBX3 gene amplification is associated to increased levels of RAC1 transcripts. **A**–**I** Charts showing that CBX3 gene amplification is coupled to a low-grade and high-grade increase in RAC1 gene copy number in (**A**) esophagus and stomach cancers, (**B**) non-small cell lung cancer, (**C**) urothelial cancer, (**D**) endometrial cancer, (**E**) brain cancer, (**F**) head and neck cancer, (**G**) breast cancer, (**H**) skin melanoma, (**I**) prostate cancer. **J**–**S** Charts showing that the samples harboring CBX3 gene amplification display increased expression of RAC1 mRNA in the (**J**) TCGA Pan Cancer Atlas cohort, (**K**) bladder cancers, (**L**) uterine cancer, (**M**) non-small cell lung cancer, (**N**) prostate cancer, (**O**) skin melanoma, (**P**) esophagus and stomach cancers, (**Q**) breast cancer, (**R**) brain cancer, (**S**) head and neck cancer. Data are expressed as mean ± SEM; *n* = samples per group. *P < 0.05; **P < 0.01; ***P < 0.001, ****P < 0.0001, ns not significant. T Student’s test unpaired (**J**–**S**).

**Fig. 4:**
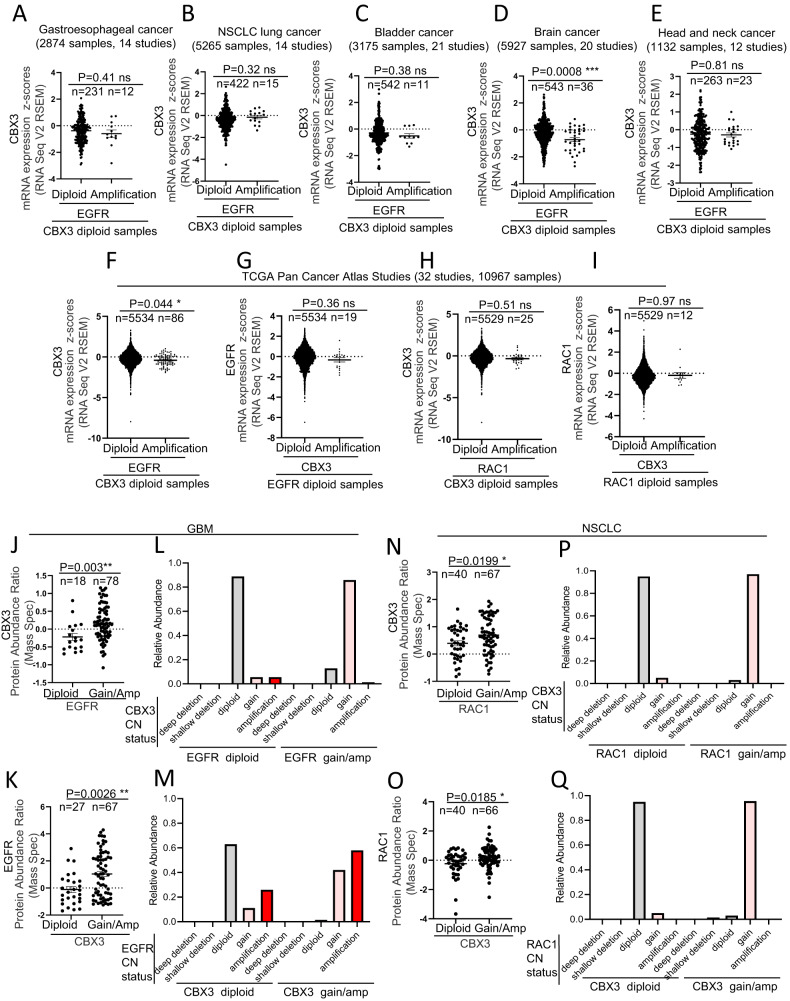
CBX3, RAC1 and EGFR gene amplification affects EGFR, RAC1 and CBX3 protein levels. **A**–**F** Charts showing that the specimens bearing EGFR gene amplified and CBX3 gene diploid do not show any significant increase in CBX3 transcriptional expression in (**A**) esophagus and stomach cancers, (**B**) non-small cell lung cancer, (**C**) urothelial cancer, (**D**) brain cancer, (**E**) head and neck cancer, (**F**) TCGA Pan Cancer Atlas patient cohort. **G** Charts showing that the TCGA Pan Cancer Atlas specimens harboring EGFR gene diploid and CBX3 locus amplified do not show any significant increase in EGFR transcriptional expression. **H** Charts showing that the TCGA Pan Cancer Atlas specimens harboring CBX3 gene diploid and RAC1 locus amplified do not show any significant increase in CBX3 transcriptional expression. **I** Charts showing that the TCGA Pan Cancer Atlas specimens harboring RAC1 gene diploid and CBX3 locus amplified do not show any significant increase in RAC1 transcriptional expression. **J**–**K** Charts showing that low- and high-grade EGFR gene amplification is accompanied by increased CBX3 protein levels (**J**) and vice versa (**K**) in glioblastoma multiforme. **L** Chart showing that the glioblastoma specimens bearing low- or high-grade EGFR gene amplification is coupled to enrichment in low-gain amplification in CBX3 gene. **M** Chart showing that the glioblastoma specimens bearing low- or high-grade CBX3 gene amplification is coupled to enrichment in low- and high-grade amplification in EGFR gene. **N**–**O** Charts showing that low- and high-grade RAC1 gene amplification is accompanied by increased CBX3 protein levels (**N**) and vice versa (**O**) in non-small cell lung cancer. **P** Chart showing that the non-small cell lung cancer specimens bearing low- or high-grade RAC1 gene amplification is coupled to enrichment in low-grade amplification in CBX3 gene. **Q** Chart showing that the glioblastoma specimens bearing low- or high-grade CBX3 gene amplification is coupled to enrichment in low-grade amplification in RAC1 gene. Data are expressed as mean ± SEM; *n* = samples per group. *P < 0.05; **P < 0.01; ***P < 0.001, ****P < 0.0001, ns not significant. T Student’s test unpaired.

We next analyzed the RAC1 CN status in the tumor samples (shown in Fig. 1), displaying either diploid or amplified CBX3 gene. We found that, similarly to what previously described for CBX3 and EGFR, whereas 90–95% of samples which are diploid for CBX3 are diploid for RAC1 (Fig. 3A–I), the tumors displaying gene amplification in CBX3 locus show a dramatic enrichment in the frequency of both low (gain) and high (amplification) CN increase of RAC1 (Fig. 3A–I), (10–40% and the 10–60% of the tumor specimens, respectively). Moreover, the tumor samples with amplified RAC1 display a drastic increase of either low gain or high-grade amplification of CBX3 gene (Supplementary Fig. 5A–I). Next, similarly to CBX3 and EGFR loci, we also observed that RAC1 gene amplification positively correlated with the upregulation of the corresponding mRNAs (Supplementary Fig. 6A–J). Interestingly, we have also found that in cancer samples where CBX3 gene is amplified, there is also a statistically significant increase of RAC1 mRNA expression regardless the tumor tissue of origin. We can thus argue that the co-occurrence of CBX3 and RAC1 gene amplification might be a way to increase RAC1 and CBX3 expression during cancer development, two well-known proteins that have been linked to cancer growth and proliferation when overexpressed (Fig. 3J–S). On the other hand, cancer samples from TCGA Pan Cancer Atlas Studies harboring high CN increase in RAC1 gene display enhanced expression of CBX3 RNAs (Supplementary Fig. 6K), thus further confirming that gene amplification of RAC1 and CBX3 leads to an increase of CBX3 and RAC1 mRNAs, respectively in human cancers. The positive and reciprocal correlation between CBX3, EGFR and RAC1 CNV and the relative gene expression prompted us to verify whether such transcriptional increase might be a consequence of the co-occurrence of gene amplification between CBX3-EGFR and RAC1-CBX3 or could result from a direct regulation of the relative gene/transcript/protein products at some other level irrespective to the gene amplification. To address this question, we analyzed CBX3 mRNA expression in the samples harboring amplified EGFR gene and/or RAC1 and 2 copies of CBX3. Interestingly, in none of the diverse cancer types analyzed, including TCGA Pan Cancer Atlas samples, we observed an increase of CBX3 transcripts compared to tumor samples which are diploid for either EGFR or RAC1 (Fig. 4A–I). This evidence indicates that the increase in CBX3 mRNA (Fig. 2J–S) is ascribable more to the expression of CBX3 co-amplified copies rather than to a regulatory effect of either EGFR or RAC1 on the transcription of CBX3. Moreover, no change in either EGFR or RAC1 transcript levels was observed in the samples with amplified CBX3 and 2 copies of EGFR or RAC1 genes, respectively (Fig. 4A–I).

### Amplification of CBX3 gene is associated with RAC1 and EGFR protein level increase and vice versa

Next, we set to verify whether the CBX3 mRNA increase in the specimens with EGFR/RAC1 amplified (and vice versa) was also accompanied by corresponding change in protein levels. We focused on GBM and lung tumors, as they are currently the only tumor types in which it has been provided evidence of an indirect CBX3-mediated regulation of EGFR [17] and RAC1 [18], respectively. Consistently to mRNA data, mass spectrometry data from GBM samples from Clinical Proteomic Tumor Analysis Consortium (CPTAC, see Materials and Methods) bearing either low- or high-grade increase of EGFR CN display a significant increase in CBX3 protein levels, and analogously, specimens with multiple copies of CBX3 gene show a statistically significant enrichment of EGFR protein levels (Fig. 4J, K). Furthermore, the analysis of the CN status of such samples revealed that the specimens with multiple copies of EGFR gene have a strong tendency to display low gain increase in CBX3 CN (80% of the samples compared to 5% of tumor with EGFR gene diploid) (Fig. 4L). Conversely, whereas the 60% of GBM samples with 2 copies of CBX3 are also normoploid for EGFR gene, all tumor samples with multiple copies of CBX3 gene display low or high gain increase of EGFR gene CN (Fig. 4M). Altogether these data suggest that the increase in transcripts as well as protein levels of both EGFR and CBX3 in the samples with multiple copies of CBX3 and EGFR genes, respectively, may be ascribable to the co-occurrence of amplification of CBX3 gene in the samples with EGFR gene amplified and vice versa.

In line with the genomic datasets, mass spectrometry data in NSCLC samples from the CPTAC (See Materials and Methods section) bearing either low or high gain of RAC1 CN reveal a significant increase of CBX3 protein levels, and analogously, specimens with multiple copies of CBX3 show a statistically significant enrichment of RAC1 protein (Fig. 4N, O). Moreover, almost all (95%) specimens bearing multiple copies of RAC1 gene have a strong tendency to display low gain increase in CBX3 CN (compared to 5% of samples with RAC1 gene diploid) (Fig. 4P). Conversely, whereas up to 95% of NSCLC samples, which are diploid for CBX3 are also normoploid for RAC1 gene, 95% of tumor samples harboring multiple copies in CBX3 gene display low gain increase in RAC1 CN (Fig. 4Q). Altogether these data provide further evidence that the increase in transcripts as well as protein levels of both RAC1 and CBX3 respectively in the samples with multiple copies of CBX3 and RAC1 genes may be a consequence of the co-occurrence of amplification of CBX3 gene in the samples with RAC1 gene amplified and vice versa.

### Gene co-amplification of CBX3 with either EGFR or RAC differentially affects patient survival

**Fig. 5:**
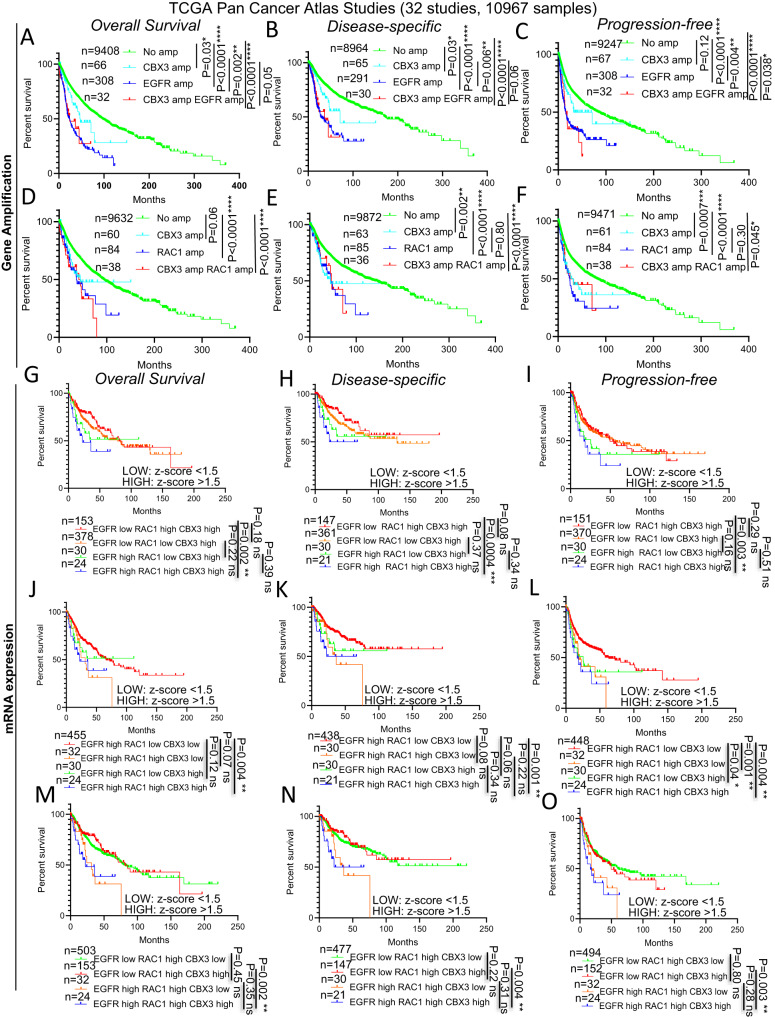
CBX3, RAC1 and EGFR overexpression and gene amplification negatively affect cancer patient lifespan. **A**–**C** Survival curves showing the (**A**) overall survival, (**B**) disease-specific survival and (**C**) progression-free survival of TCGA Pan Cancer Atlas patients harboring concomitant or single high-grade amplification in CBX3, EGFR genes. **D**–**F** Survival curves showing the (**D**) overall survival, (**E**) disease-specific survival and (**F**) progression-free survival of TCGA Pan Cancer Atlas patients harboring concomitant or single high-grade amplification in CBX3, RAC1 genes. **G**–**I** Survival curves showing the (**G**) overall survival, (**H**) disease-specific survival and (**I**) progression-free survival of TCGA Pan Cancer Atlas patients displaying high expression of CBX3 compared to the specimens having, in addition to high mRNA levels of CBX3, either EGFR or RAC1 highly expressed, singularly or in combination. **J**–**L** Survival curves showing the (**J**) overall survival, (**K**) disease-specific survival and (**L**) progression-free survival of TCGA Pan Cancer Atlas patients displaying high expression of EGFR compared to the specimens having, in addition to high mRNA levels of EGFR, either CBX3 or RAC1 highly expressed, singularly or in combination. **M**–**O** Survival curves showing the (**M**) overall survival, (**N**) disease-specific survival and (**O**) progression-free survival of TCGA Pan Cancer Atlas patients displaying high expression of RAC1 compared to the specimens having, in addition to high mRNA levels of RAC1, either CBX3 or EGFR highly expressed, singularly or in combination. *n*= samples per group. *P < 0.05; **P < 0.01; ***P < 0.001, ****P < 0.0001, ns = not significant. Z-score values higher than 1.5 were considered as being “high expression”. Logrank test (**A**–**F**) and Gehan-Breslow-Wilcoxon test (**G**–**O**).

Next, we set out to check whether the co-amplification of CBX3 with either EGFR or RAC1 genes might affect cancer aggressiveness and patient lifespan. To this end, by using the data from the TCGA Pan Cancer Atlas, we first found that CBX3 gene amplification negatively correlates with patient survival (Supplementary Fig. 7A–C). To understand whether the poor prognosis associated with CBX3 gene amplification might result from to the co-amplification of either EGFR or RAC1 loci, we first plotted the survival curves on the basis of the CN status of both CBX3 and EGFR genes. This analysis revealed that, albeit the patients with amplification of CBX3 CNs tend to show a shorter lifespan compared to patients diploid for both CBX3 and EGFR (Fig. 5A–C), the overall survival (OS), disease-specific (DS) as well as the progression-free (PF) curves displayed significantly shortened survival of patients showing EGFR gene amplification in comparison to those having CBX3 amplified (Fig. 5A–C). Moreover, the patients harboring multiple copies of both EGFR and CBX3 genes display survival curves comparable to those having only the EGFR locus amplified and consequently show a tendency to have a poorer prognosis compared to the cohorts displaying tumors with only CBX3 gene amplified. Importantly, such trend reaches the statistical significance in the PF survival (Fig. 5C), therefore suggesting that the shorter PF survival observed in patients with CBX3 gene amplification (Supplementary Fig. 7C) may be due, at least in part, to the amplification of EGFR gene which co-occurs with CBX3 CN increase.

We have also found no significant difference in lifespan reduction among patients harboring amplification of either CBX3 or RAC1 only, and patients harboring co-amplification of CBX3 and RAC1 (Fig. 5D–F). This rules out that the presence of co-amplification of CBX3 and RAC1 genes per se could affect the OS, PF, DS curves. Finally, we also observed that while CBX3 gene amplification is co-analyzed with EGFR locus amplification it does not have any impact on the PF survival (Fig. 5C), when co-analyzed with RAC1 high-grade CBX3 amplification results in a significant reduction in life expectancy (Fig. 5F), thus enforcing the evidence that the association between CBX3 amplification and poor patient prognosis is attributable, at least partially, to the co-occurring amplification of EGFR gene.

### Simultaneous overexpression of CBX3, EGFR and RAC1 negatively correlates with cancer patient survival

We set out to understand whether an increased gene expression of CBX3 with either EGFR or RAC1 could also result in a poor prognosis. To this end, we plotted the survival curves by organizing the patients from TCGA Pan Cancer Atlas on the basis of mRNA expression and considered a z-score greater than 1.5 as an indicator of high level of gene expression. As shown in Fig. 5G–L, we found that EGFR overexpression in the patients with high levels of CBX3 correlates with a statistically significant worsening of OS, DS and PF when also RAC1 is overexpressed (Fig. 5G–I), thus potentially indicating that EGFR may synergize with RAC1 in the worsening of the survival of the cohort with high levels of CBX3. Conversely, in the cohort displaying highly expressed EGFR, the patients with either RAC1 or CBX3 overexpressed tend to display a worse outcome, which reaches statistical significance in PF curve, thus indicating a potential functional interaction of EGFR with both CBX3 and RAC1 (Fig. 5J–L). Coherently with that, the strongest effect on patient survival is observed when concomitant high levels of both CBX3 and RAC1 are associated with EGFR overexpression (Fig. 5J–L). Collectively, these findings indicate that the simultaneous overexpression of CBX3, RAC1 and EGFR could lead to a negative synergistic effect on cancer patient survival.

Conversely, in line with survival analysis shown in Fig. 4D–F, a simultaneous increase of the expression of RAC1 and CBX3 mRNAs does not correlate with severe effects on either OS, DS or PF survival compared to the patients showing high levels of either CBX3 or RAC1 transcripts alone, regardless the status of EGFR expression (Fig. 5M–O). Moreover, in all the curves, high levels of EGFR negatively correlate with patient survival in the presence of RAC1 overexpression. These observations might suggest that CBX3 and RAC1 may play redundant functions in cancer and indicate a potential functional interaction between EGFR and RAC1.

Importantly, the analysis of EGFR and CBX3 CN status in patients displaying simultaneous high expression of CBX3 and EGFR revealed the existence of a drastic reduction in diploid samples and a concurrent 5- and 2.25- fold increase in high-grade amplification in both EGFR and CBX3 compared to the patients showing high expression of only CBX3 or EGFR, respectively (Supplementary Fig. 8A–E). Therefore, albeit the concomitant high-grade amplification of such genes occurs only in about 4% of the patients displaying high levels of CBX3 and EGFR mRNAs, it is reasonable that it may contribute, at least partially, to the induction of concomitant upregulation of EGFR and CBX3 expression (Supplementary Fig. 8A–E). Conversely, almost the 40% of the cohort with concomitant high levels of CBX3 and EGFR mRNAs show simultaneous high-grade amplification of EGFR and low-gain increase in CBX3, which is 2- and 17-fold more represented compared to the patients displaying high levels in either CBX3 or EGFR respectively. This suggests that the concurrent high-copy increase of EGFR and low-copy increase in CBX3 genes may play a significant role, in combination with potential additional mechanisms, in promoting higher expression of both EGFR and CBX3 (Supplementary Fig. 8A–E).

### Low-grade copy number variations (CNVs) in CBX3 locus co-occur with those in EGFR and RAC1 genes

**Fig. 6:**
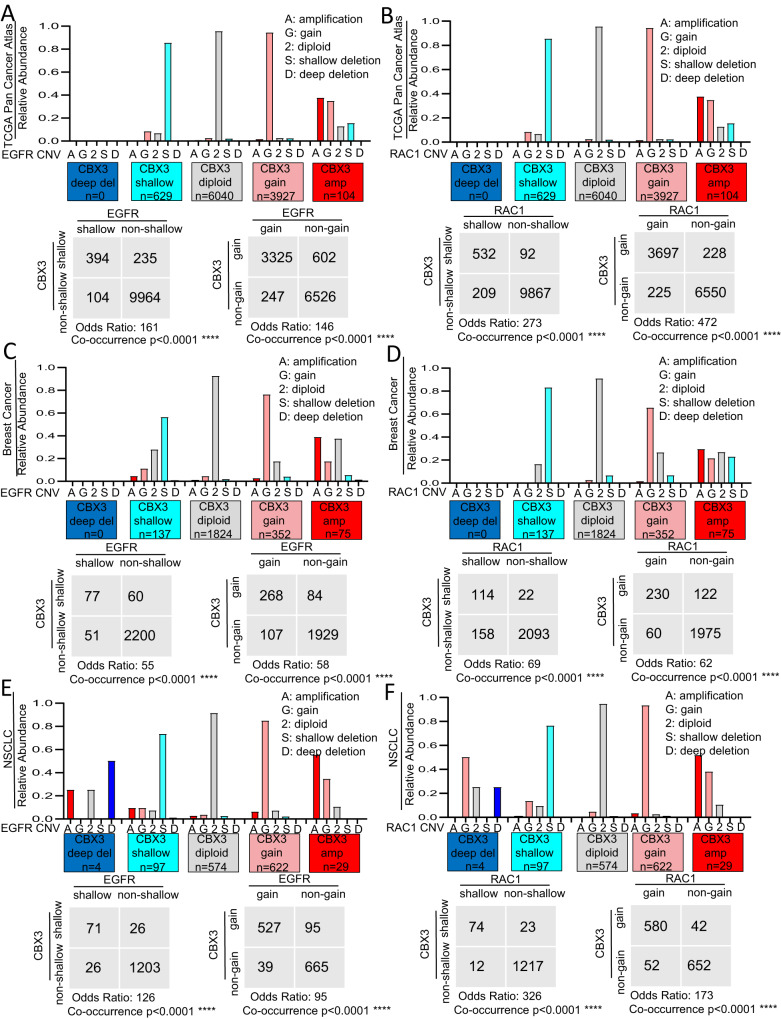
Low-grade copy number alterations in CBX3, EGFR and RAC1 genes co-occur in human cancer. Charts (up) and contingency tables (bottom) respectively showing the overall copy number status and co-occurrence of shallow deletions as well as low-level gain in gene CN of CBX3 with either EGFR (**A**, **C**, **E**) or RAC1 (**B**, **D**, **F**) in the TCGA Pan Cancer Atlas patient cohort (**A**, **B**), breast cancer (**C**, **D**) and non-small cell lung cancer (**E**, **F**). *n* = number of samples. *P < 0.05; **P < 0.01; ***P < 0.001, ****P < 0.0001, ns = not significant. Fisher’s exact test.

We next observed that, in TCGA Pan Cancer Atlas as well as in breast, lung, brain, prostate and skin cancer patients (see Materials and Methods) in addition to high-grade gene amplification, also low-grade variations (i.e. low-copy gain and shallow deletions) in CBX3 gene strongly co-occur with low-grade variations of either EGFR or RAC1 genes (Fig. 6A–F; Supplementary Fig. 9A–F). Importantly, the co-occurrence in either gene low-copy gain or shallow deletions of CBX3 locus with EGFR and RAC1 genes is a rather frequent genetic event occurring in about 35% and 40% of the TCGA Pan Cancer Atlas cohort, respectively (Supplementary Fig. 10A, B). Furthermore, in the same cohort we also observed that while high-grade amplifications in CBX3 gene co-occurred with shallow deletions in both EGFR and RAC1 genes, shallow deletions in CBX3 co-occurred with low- and high-grade amplifications for both EGFR and RAC1 genes, thus enforcing the evidence that CNAs in CBX3 are associated with general perturbations (high- and/or low-grade amplifications) in the CN of EGFR and RAC1 loci (Supplementary Fig. 10C–F). Finally, we also found that whereas low copy gain and shallow deletions in both CBX3 and RAC1 genes are associated to a statistically significant upregulation and down-regulation in the expression of the corresponding mRNAs, respectively, (Supplementary Figs. 3A–J and 6A–J), CNVs in EGFR gene are associated not always with alterations of EGFR transcripts (Supplementary Fig. 4A–J). Taken together these data suggest that low-grade CNVs in CBX3, EGFR and RAC1 loci strongly co-occur in tumors and with the exception of EGFR, could contribute to increase the expression of the corresponding genes.

### Low copy gain and shallow deletions in CBX3, EGFR and RAC1 genes negatively correlate with patient survival

**Fig. 7:**
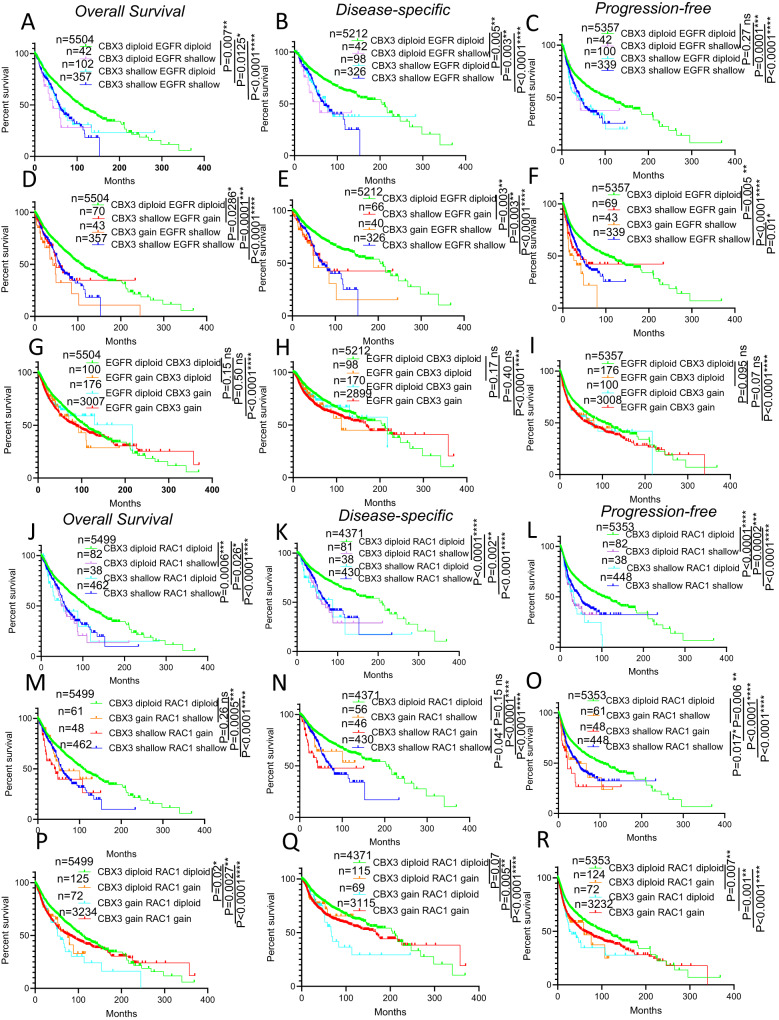
Low-grade copy number variations in CBX3, RAC1 and EGFR genes negatively affect cancer patient lifespan in TCGA Pan Cancer Atlas cohort. **A**–**C** Survival curves showing the (**A**) overall survival, (**B**) disease-specific survival and (**C**) progression-free survival patients harboring concomitant or single shallow deletions in CBX3 and EGFR genes. **D**–**F** Survival curves showing the (**D**) overall survival, (**E**) disease-specific survival and (**F**) progression-free survival of patients harboring concomitant shallow deletions in CBX3 and EGFR genes versus specimens bearing shallow deletions in CBX3 and low-level copy gain in EGFR and vice versa. **G**–**I** Survival curves showing the (**G**) overall survival, (**H**) disease-specific survival and (**I**) progression-free survival of patients displaying concomitant or single low-level copy gain in CBX3 and EGFR genes. **J**–**L** Survival curves showing the (**J**) overall survival, (**K**) disease-specific survival and (**L**) progression-free survival patients harboring concomitant or single shallow deletions in CBX3 and RAC1 genes. **M**–**O** Survival curves showing the (**M**) overall survival, (**N**) disease-specific survival and (**O**) progression-free survival of patients harboring concomitant shallow deletions in CBX3 and RAC1 genes versus specimens bearing shallow deletions in CBX3 and low-level copy gain in RAC1 and vice versa. **P**–**R** Survival curves showing the (**P**) overall survival, (**Q**) disease-specific survival and (**R**) progression-free survival of patients displaying concomitant or single low-level copy gain in CBX3 and RAC1 genes. *n* = samples per group. *P < 0.05; **P < 0.01; ***P < 0.001, ****P < 0.0001, ns not significant. Logrank test (**A**–**R**).

We next asked whether low-grade variations in CBX3, EGFR and RAC1 gene could affect patient lifespan. Shallow deletions in either CBX3 or EGFR are associated with a shorter patients’ lifespan compared to the diploid counterparts (Fig. 7A–C). However, a concomitant shallow deletion of CBX3 and EGFR genes is not correlated with a more severe prognosis compared to that of patients harboring single shallow deletions (Fig. 7A–C). Moreover, the presence of a low copy gain in one of two genes with a shallow deletion in the other does not have any significant impact on the OS and DS curves. Nevertheless, patients with a low-gain increase in CBX3 locus and a shallowly deleted EGFR gene, exhibited PF survival significantly shorter than patients with both CBX3 and EGFR shallowly deleted (Fig. 7D–F). Furthermore, even though low gain in CNs in either CBX3 or EGFR gene alone does not affect patient lifespan, patients with a concomitant low copy gain in both CBX3 and EGFR genes show a slight, yet statistically significant, worse OS, DS and PF survivals compared to those with two normal copies of CBX3 and EGFR genes (Fig. 7G–I). Collectively these observations suggest a potential functional interaction of subtle CNV of EGFR and CBX3 genes that could affect patients’ lifespan. Finally, it is noteworthy to point out that CBX3 gene low copy gain correlates with a worsening in PF survival only in patients showing EGFR shallowly deleted (Fig. 7F) but not when EGFR is diploid (Fig. 7I), thus arguing that only specific combinations of low-grade CNV in CBX3 and EGFR loci may result in a synergistic effect that leads to reduction of lifespan.

Concurrent or single shallow losses in either CBX3 or RAC1 genes also result in a statistically significant shortening of lifespan compared to the diploid counterpart (Fig. 7J–L). However, the presence of low copy gain in CBX3 locus in patients with shallow deletions of RAC1 gene, but not the reciprocal, correlates with a reduction of OS and DS curve shortening normally associated with the presence of RAC1 gene shallow deletions (Fig. 7M, N). Conversely, the presence of low copy gain in RAC1 gene correlates with a further worsening of both DS and PF life expectancy with respect to patients with both shallowly deleted RAC1 and CBX3 genes (Fig. 7N, O). This suggests that low copy gain in either CBX3 or RAC1 gene, has an opposite effect on patient lifespan when combined with the shallow deletion of RAC1 or CBX3, respectively. These findings, coupled to the evidence that shallow deletions in CBX3 gene are significantly associated to low copy gain in RAC1 gene (Supplementary Fig. 10F), argue for the possibility that this specific combination in CNAs between CBX3 and RAC1 loci may be indeed positively selected during cancer development to confer an evolutionary advantage to transformed cells.

Furthermore, patients with concurrent low copy gain in RAC1 and CBX3 genes display a significant lower lifespan compared to patients diploid for both genes (Fig. 7P–Q). Yet, differently from EGFR, low gene copy increase of either single RAC1 or CBX3, or of RAC1 CBX3 combination, is associated with a negative effect on patient lifespan (Fig. 7P–Q). Although such data might suggest that the ability of a given subtle CNV to affect patient survival is context-dependent, the poor prognosis observed in the patients with low copy gain and two copies of CBX3 and RAC1 genes, respectively may be potentially due to the co-occurrence of low gain in CBX3 gene copies with EGFR gene amplification, which is known to result in worse prognosis (Figs. 2A–I, 5A–C, 6A).

### Genetic analysis in *Drosophila melanogaster* reveals a conserved functional relationship among CBX3, EGFR and RAC1

**Fig. 8:**
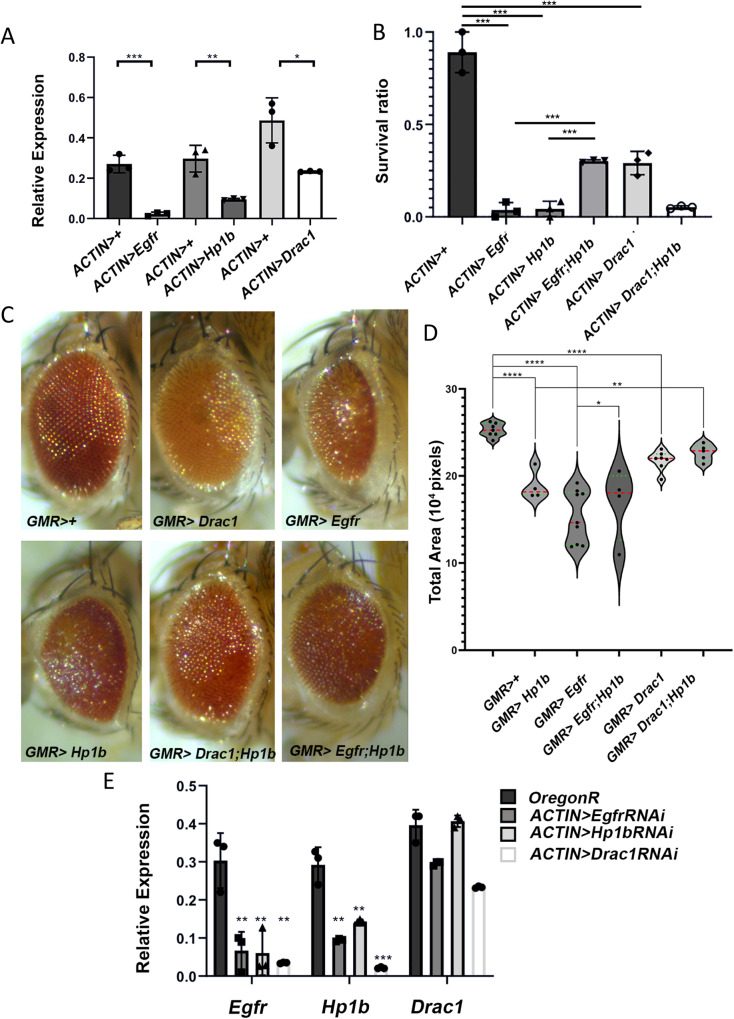
Drosophila Hp1b genetically interacts with either Egfr or dRac1. **A** Reduced expression of *Egfr*, *Hp1b* and *dRac1* mRNAs in the corresponding interfered flies, analyzed by qPCR. Three different replicates were performed for this analysis (*p < 0.05; **p < 0.01; ***p < 0.001 Student’s *t*-test). **B** Effects of ubiquitous depletion of *Egfr, Hp1b* and *dRac1* or *Egfr; Hp1b* and *dRac1;Hp1b* on flies’ viability. Note that simultaneous depletion of *Egfr* and *Hp1b* robustly alleviates the lethality of single *Egfr* and *Hp1b* interfered flies (***p < 0.001 two-way ANOVA test). **C** *GMR****-****GAL4/UASdsRNA* mediated depletion of *Drac1 (GMR>dRac1), Egfr (GMR > Egfr)* and *Hp1b (GMR* > *Hp1b)* specifically in the Drosophila eye results in a rough-like phenotype and reduced eye size, although with different severity. Note that depletions of Hp1b and Rac1 alleviate both rough eye and reduced size phenotypes of *GMR>Egfr* and *GMR* > *Hp1b* flies, respectively. **D** Violine plot showing the differences in eye area among the different RNA interfered flies. Bottom green line: first quartile (Q1); top green line: third quartile (Q3); red line: median (Q2). All comparisons were performed using ANOVA test: statistically significant *p < 0.05; **p < 0.01; ***p < 0.001; ****p < 0.0001. **E** Effects of ubiquitous depletion of *Egfr, Hp1b* or *dRac1* on the expression of single *Egfr*, *Hp1b* and *dRac1* mRNAs revealed by qPCR. Three different replicates were performed (**p < 0.01; ***p < 0.001, ANOVA test).

We set out to understand whether the correlation between co-occurring gene amplification of CBX3, RAC1 and EGFR and severe patient prognosis underlies a functional relationship among these three genes. We leveraged Drosophila sophisticated genetics to verify whether the Drosophila ortholog of CBX3, *HP1b* genetically interacted with *Egfr* and *Rac1* genes that encode the fly orthologs of human EGFR and RAC1, respectively. By using the well-established GAL4-UAS binary system and RNAi lines from VDRC, we knocked down *Hp1b, Egfr* and *Rac1* transcripts in transgenic flies expressing shRNAs directed against mRNAs from each one of three genes (Fig. 8A). Consistent with previous works, *actin Gal4*-dependent ubiquitous RNAi induction for each gene resulted in lethality of *actin Gal4* > *UAS Hp1bRNAi*, *actin Gal4* > *UAS egfrRNAi* and *actin Gal4* > *UAS rac1RNAi* third instar larvae confirming that all genes play a fundamental role during fly development [22–24]. Interestingly, a simultaneous depletion of *Hp1b* and *Egfr (*but not of *Hp1b* and *Rac1)* yielded few *actin Gal4* > *UAS Hp1bRNAi; UAS egfrRNAi* viable adult escapers (Fig. 8B) indicating that loss of Hp1B can partially suppress the lethality caused by the depletion of Egfr or vice versa thus providing a compelling evidence of a genetic interaction. To further confirm these results, we asked whether a similar suppression was also evident for the eye phenotypes induced by a *GMR-Gal4-*driven RNAi in the eye. RNAi in the larval eye disc for all three genes resulted in an altered morphology of adult eye (Fig. 8C). Consistently with previous results [25], *GMR-Gal4* > *UAS efgrRNAi* flies exhibited a strong reduction of eye size (Fig. 8C, D). Eye morphology and size of *GMR-Gal4* > *UAS Hp1bRNAi* and *GMR-Gal4* > *UAS rac1RNAi* flies were also affected as a consequence of the depletion of the corresponding genes, although the phenotypes are not as strong as in *egfr* depleted flies. Interestingly, the eye phenotypes of both *egfrRNAi* and *rac1RNAi* flies was partially suppressed by the knockdown of *Hp1b*, thus confirming that *Egfr* genetically interact with *Hp1b* and indicating that, at least in this genetic set up, *Rac1* also interacts with *Hp1b* (Fig. 8C, D). We have also checked whether the mRNA expression of *Egfr* and/or *Rac1* was influenced by *Hp1b*. Interestingly, qPCRs from RNAi lines revealed that not only *Egfr* and *Rac1* mRNA levels decreased upon depletion of *Hp1b*, but also depletion of either *Egfr* or *Rac1* resulted in a reduction of *Hp1b* mRNA expression (Fig. 8E). These results indicate for the first time that the transcript levels of *Egfr, Hp1b* and *Rac1* are interdependent providing further evidence of reciprocal genetic interactions.

## Discussion

In this report, we showed for the first time that CBX3 gene amplification strongly co-occurs with both EGFR and RAC1 genes in different human cancers and that this molecular event is associated with an increase of mRNA and protein levels of CBX3, EGFR and RAC1. We have also found that the high-grade amplification of either CBX3, RAC1 or EGFR is associated with a drastic increase in low copy gain of the other two genes.

The evidence that this gene co-amplification occurs in different human cancers could suggest a functional relationship between CBX3, EGFR and RAC1 genes which normally occurs in physiological conditions but that could promote cancer progression when dysregulated. However, although we revealed that the poor prognosis of the patients harboring CBX3 gene amplified in part correlates with the co-amplification of EGFR gene, the patients with amplified EGFR and CBX3 show a lifespan comparable to that of patients with amplified EGFR only (see below). On the other hand, we also uncovered that the simultaneous overexpression of CBX3, RAC1 and EGFR gene products is associated with a statistically significant worse prognosis compared to the condition in which CBX3, RAC1 and EGFR are upregulated singularly or in pairs, therefore suggesting that a clear combined effect of the overexpression of both CBX3, RAC1 and EGFR on the worsening of patient survival can be observed only when these genes are dramatically upregulated. These findings let us speculate the existence of a potential functional relationship between EGFR and RAC1 and enforce our evidence of a deep interconnection between EGFR and CBX3. Thus, at least for EGFR and CBX3, it is conceivable that the concurrent presence of multiple copies of EGFR and CBX3 genes might not be the most efficient/common way to accomplish the highest extent of simultaneous overexpression of both EGFR and CBX3 and consequently it might prevent from observing a clear association between the expression of these genes and patient survival. Consistently, we unveiled that the majority of the cancer samples concomitantly expressing the highest levels of CBX3 and EGFR specifically show higher occurrence in EGFR high-grade amplification coupled to low-gain increase in CBX3 gene CN. This observation lets us envisage that the simultaneous low and high-grade copy gain increase in CBX3 and EGFR genes respectively, may cooperate or may be coupled with additional mechanisms to achieve productive upregulation of these cancer-promoting genes which in turn results in a dramatic effect on cancer patient life expectancy. Collectively, our findings indicate that the concomitant overexpression of both EGFR, CBX3 and RAC1 may be an extremely unfavorable general prognosis marker in human cancer.

We also unveiled that in addition to co-occurrence of gene amplification among CBX3, EGFR and RAC1, also low-grade CNVs of these genes strongly co-occur in several human tumor types and are much more recurrent than high-grade CNVs. Intriguingly, in contrast to what observed for the concomitant high-grade gene amplification of CBX3 with either EGFR or RAC1, the frequency of either simultaneous shallow deletions or low-gain copy increase in CBX3 with either EGFR or RAC1 is much higher than the occurrence of such low-grade CNVs encompassing only one of these genes. We uncovered that shallow deletions as well as low-copy gain increase occurring in CBX3 with either EGFR or RAC1 singularly or simultaneously are associated with a poor prognosis in the TCGA cohort. These findings, coupled to the high frequency in the co-occurrence of either shallow deletions or low-copy gain in EGFR, CBX3, RAC1 genes, let us speculate that low-grade CNVs may be used as novel prognostic marker in human cancer. How subtle changes in CNVs may significantly affect the prognosis it is yet to be elucidated. In many tumor types shallow deletion and low-level copy gain for CBX3 and RAC1, and in a lesser extend for EGFR, results in a proportional manner respectively significantly lower and higher mRNA levels of the corresponding genes. Therefore, we do not rule out the possibility that these subtle changes in protein/mRNA expression might contribute, at least in part, to such a poor outcome. Nevertheless, a more plausible scenario is that such CNV changes might be associated and/or facilitate further changes in other oncogenes and tumor-suppressors or may be specifically associated to more aggressive cancer types or subtypes. In support of this hypothesis, it has been recently reported that the impact of a mutation/CNA of a oncogene or tumor-suppressor gene on cancer development is not a universal characteristic but is context-dependent and relies on the presence of other CNAs and mutations occurring in other genes throughout the genome [26]. The analysis of specific combinations in these low-grade CNVs, has also disclosed a potential functional interaction between CBX3 and RAC1. Indeed, the presence of shallow deletions in CBX3 genes coupled to low-copy gain increase of RAC1 locus not only negatively affects the DS and PF survival compared to diploid patients, but results in a further worsening of life expectancy compared even to the cohort displaying shallow deletions in both genes. Conversely, although the patients harboring RAC1 locus shallowly deleted show poor outcome, the presence of low-copy gain in CBX3 locus in this genetic background restores OS as well as DS survival to levels comparable to those of patients bearing both the genes diploid.

Altogether, these observations potentially suggest that the impact of low-grade CNVs in CBX3 and RAC1 genes on patient lifespan may be not unspecific but dictated on the basis of which of the two genes displays a low-grade copy increase and the other the shallow deletion. This “directionality” may reflect the existence of a functional interaction between CBX3 and RAC1, which is sensitive to specific combinations of subtle CNVs in CBX3 and RAC1 genes. Thus, although further studies are required for the validation of this working hypothesis in specific tumor types as well as for the identification of the mechanism underlying this functional relationship, here we have identified specific combinations of low-grade CNVs as a novel and potential prognosis markers in human cancer. Finally, we have shown for the first time in vivo that CBX3 genetically interacts with both EGFR and RAC1 in *Drosophila melanogaster*, thus suggesting that the simultaneous overexpression as well as gene co-amplification events among these genes are not accidental but reflect an evolutionarily conserved functional relationship among these genes.

## Materials and Methods

### Drosophila stocks and genetics

The RNAi Drosophila transgenic lines expressing UAS-driven dsRNAs for *Drac1 (v49247), egfr (v43268)*, and *Hp1b (v26097)* were obtained from Vienna Drosophila Research Center (VDRC). Vectors encoding *UAS rac1 dsRNA, UAS egfr dsRNA* and *UAS Hp1b dsRNA* are inserted on chromosomes X, 2 and 3, respectively. The *UAS rac1 dsRNA* insertion was maintained in a stock containing *UAS rac1 dsRNA/Y* males and compound *yws^yws/Y* females. Single *egfr* or *Hp1b* RNAi-induced phenotype (*actin*- and/or *GMR Gal4* > *UAS egfr dsRNA* and *actin*- and/or *GMR Gal4* > *UAS Hp1b dsRNA)* was obtained in the progeny from crossing *actin Gal4/TM6b* or *GMR Gal4/CyO* flies to each *UAS dsRNA* line and selecting larvae/adults that did not carry the *TM6b* or the *CyO* balancers for *actin Gal4-* and *GMR Gal4* mediated expression, respectively*. actin*- and/or *GMR Gal4* > *UAS rac1 dsRNA* was obtained upon crossing *UAS rac1 dsRNA/Y* males to either *actin Gal4/TM6b* or *GMR Gal4/CyO* females and selecting only female larvae/adult progeny that did not carry the *TM6b* or the *CyO* balancers. To obtain double RNAi for *egfr* and *Hp1b, UAS egfr dsRNA; UAS Hp1b dsRNA/TSTL* flies were crossed to *actin Gal4/TM6b* flies and progeny that did not carry the TSTL balancer selected for the analysis. Double RNAi for *Rac1* and *Hp1b* was obtained by crossing *UAS Rac1 dsRNA/Y; UAS Hp1b dsRNA/TM6b* males to either *actin Gal4/TM6b* or *GMR Gal4/CyO* females and selecting only female larvae/adult progeny that did not carry the balancers. All strains were maintained at 25 °C on *Drosophila* medium (Nutri-Fly®GF; Genesee Scientific) treated with propionic acid. The detailed information on the balancers and the genetic markers used are available online on Flybase (http://flybase.bio.indiana.edu/).

### Screening of fly lethality

Lethality test was performed by analyzing the progeny of *Actin Gal4/TM6b* males to *UAS RNAi* homozygous females. Adult lethality was calculated as the ratio of the number of viable *Actin Gal4; UAS RNAi (Tb* + *, Hu* + *)* adults over the number of Actin *Gal 4 TM6b (Tb, Hu)* adults (control). Statistical significance was evaluated by a Two-way Anova test.

### *Drosophila* eye analysis

Adult eyes images were taken with a Zeiss Stemi 508 stereo microscope equipped with an Axiocam camera and acquisition Zen program. Image processing and eye dimension measurements were performed with ImageJ [27] through manually eye area selection and surface measurement. Statistical significance was evaluated with Student’s t-test.

### RNA extraction and Real Time qPCRs

Drosophila RNA isolation and Real Time qPCR were carried out as previously described [28–30]. Briefly, total Drosophila RNA was isolated from third instar larvae (10 larvae/sample) using TRIzol (TRI Reagent^®^ SIGMA Life Science, Sigma-Aldrich) and genomic DNA was eliminated with Invitrogen^TM^ Dnase I Amplification Grade (Thermo Fisher Scientific) by following the manufacturer’s manual. cDNA was synthesized from 300 ng of total RNA for each sample by using the iScript™ cDNA Synthesis Kit (Bio-Rad, Hercules, CA, USA). Thirty nanograms of cDNA per reaction were analyzed for semi-qPCR using the SsoAdvanced™ Universal SYBR® Green Supermix Kit (Bio-Rad) following the manufacturer’s protocol. Real Time PCR was then performed with Quant Studio 3 Real Time PCR system using the following primers couples:

GAPDH FW 5’-GACGAAATCAAGGCTAAGGT-3’

GAPDH RV 5’-AATGGGTGTCGCTGAAGAAGTC-3’

EGFR FW 5’-TGCATCGGCACTAAATCTCGG-3’

EGFR RV 5’-GGAAGCTGAGGTCCAAATTCTC-3’

RAC1 FW 5’-GCTGATCAGCTACACGACCA-3’

RAC1 RV 5’-TGGCCGAGTAGTTGTCGAAC-3’

HP1b FW 5’-TCCGCGCAGCGAAAACACCT-3’

HP1b RV 5’-TACCATTGCCGCTGCCCGTG-3’

PCR reactions were carried out in the ABI Prism 7300 System (Applied Biosystems, Foster City, CA, USA). Data processing was performed using the ABI SDS v2.1 software (Applied Biosystems). The critical threshold value was noted for each transcript and normalized to the internal control. The fold change was calculated using the comparative 2^(−ΔΔCt)^ method.

### Bioinformatic databases and cancer studies

Copy number, mRNA expression data as well as survival curve data were obtained by querying cBioPortal [31, 32] (https://www.cbioportal.org/), GDC Data Portal (https://portal.gdc.cancer.gov/) and TCGA Pan Cancer Atlas [33] (https://www.cancer.gov/about-nci/organization/ccg/research/structural-genomics/tcga). The cancer studies analyzed for each tumor type are listed in the Supplemental File “Cancer Studies List”.

### Copy number analysis and oncoprints

Copy number data sets within the portal were generated by the GISTIC2 algorithm. Such an algorithm attempts to identify significantly altered regions of amplification or deletion across sets of patients. This algorithm also generates putative gene/patient copy number specific calls, which are then input into the cBioportal. “Amplification”, “Gain”, “Diploid”, “Shallow Deletion” and “Deep Deletion” respectively correspond to “2”, “1”, “0”, “-1”, and “-2” values which are derived from copy-number analysis algorithm GISTIC2, and indicate the copy-number level per gene: -2 or Deep Deletion indicates a deep loss, possibly a homozygous deletion; -1 or Shallow Deletion indicates a shallow loss, possibly a heterozygous deletion; 0 is diploid; 1 or Gain indicates a low-level gain (a few additional copies, often broad); 2 or Amplification indicate a high-level amplification (more copies, often focal). The oncoprints were obtained from cBioportal upon querying the database specifically for the copy number alterations for CBX3, EGFR and RAC1 genes in the samples from the analyzed tumor types.

### Aneuploidy score and fraction genome altered analysis

The values corresponding to the aneuploidy score and to the fraction of altered genome in the TGCA Pan Cancer Atlas patient cohort are available in Supplementary Materials “Raw Data TCGA PanCancer Atlas Samples” and have been downloaded from cBioportal. The aneuploidy score reflects cancer aneuploidy and has been previously described and determined for TGCA Pan Cancer Atlas patient samples [34]. The fraction of genome altered is the percentage of genome that has been affected by copy number gains or losses and has been previously described [35] and determined for TGCA Pan Cancer Atlas samples.

### mRNA expression analysis

For mRNA expression data, the raw data tables containing the Z-scores mRNA expression values of RNASeqV2 (available in Supplementary Materials “Raw Data Cancer Samples”) were downloaded from cBioportal or TCGA databases. As described in cBioportal and TCGA databases, the relative expression of an individual gene in a tumor sample to the gene’s expression distribution is computed in a reference population of samples. We selected as a reference population all profiled samples (by default for mRNA). The returned value indicates the number of standard deviations away from the mean of expression in the reference population (Z-score). All the RNA seq data showed in our study come from the RNASeqV2 mRNA expression data for normal samples of 16 TCGA PanCan Atlas Cohorts. The data were curated from GDC (https://gdc.cancer.gov/about-data/publications/pancanatlas). RNASeqV2 from TCGA is processed and normalized using RSEM [36]. Specifically, the RNASeqV2 data in cBioPortal correspond to the rsem.genes.normalized_results file from TCGA. cBioPortal then calculates z-scores as described above.

### Protein expression analysis

Mass spectrometric data for the protein expression analysis were obtained from Glioblastoma [37] and Non-small cell lung cancer [38] Clinical Proteomic Tumor Analysis Consortium (CPTAC) studies (https://proteomics.cancer.gov/programs/cptac). The raw data of samples analyzed are available in Supplementary Materials “Raw Data Cancer Samples”.

### Survival analysis

For the survival analyses, the raw data (Available in Supplementary Materials “Raw data TCGA PanCancer Atlas Samples”) were obtained from the TCGA PanCancer Atlas [33] cancer patient cohort by querying cBioportal database. OS STATUS means overall survival status (“0” -> “living” or “1” -> “deceased”) and OS MONTHS indicates the number of months from time of diagnosis to time of death or last follow up. PFS refers to “progression free survival”, indicating whether patient’s disease has recurred/progressed (PFS STATUS), and at what time the disease recurred or the patient was last seen (PFS MONTHS). The survival curves as well as the statistical analysis were obtained by using GraphPad/PRISM8.3.0 [39].

### Statistical analysis

Statistical analysis was performed using Microsoft® Excel 2016 and GraphPad/PRISM8.3.0. LogRank test was used for the statistical analysis of patient survival. Where specifically indicated, also the Gehan-Breslow-Wilcoxon test was used in patient survival analysis. Fisher’s exact test was used for the co-occurrence analysis of CNAs among CBX3, RAC1 and EGFR. Unpaired T-Student’s test or One-way ANOVA for the comparison of respectively two or more groups were used for the statistical analysis of the mRNA and protein expression data as well as for the aneuploidy score and fraction altered genome analysis. *P*-values of less than 0.05 were considered significant. *p < 0.05, **p < 0.01, ***p < 0.001, ****p < 0.0001. For experiments in Drosophila no blinding/randomization was done/used. The number of fruit flies per each experiment as well as the size of the experiments were obtained by performing power analysis. Each experiment was replicated at least 3 times.

### Supplementary information

Supplementary Figure Legends

Supplementary Figure 1

Supplementary Figure 2

Supplementary Figure 3

Supplementary Figure 4

Supplementary Figure 5

Supplementary Figure 6

Supplementary Figure 7

Supplementary Figure 8

Supplementary Figure 9

Supplementary Figure 10

Cancer Studies List

Raw data Cancer Samples

Raw data TCGA PanCancer Atlas Samples

## Data Availability

The datasets and other information that support the findings of this study are available from the corresponding author upon reasonable request.
