# Supplementary material for: Co-amplification of CBX3 with EGFR or RAC1 in human cancers corroborated by a conserved genetic interaction among the genes
Source: Cell Death Discov. 2023 Aug 26;9:317. doi: 10.1038/s41420-023-01598-5 (PMC10460438; doi:10.1038/s41420-023-01598-5)
Supplement: Supplementary file 1 — Supplementary Figure Legends [file 41420_2023_1598_MOESM1_ESM.docx]

**Co-amplification of CBX3 with EGFR or RAC1 in human cancers corroborated by a conserved genetic interaction among the genes.**

Giuseppe Bosso^1,3*^, Francesca Cipressa^2^, Liliana Tullo^3^, and Giovanni Cenci^3*^

**Short title:** Identification of novel evolutionarily conserved functional interactions among CBX3, EGFR and RAC1 in cancer

^1^ Present address: Telomeres and Telomerase Group, Molecular Oncology Program, Spanish National Cancer Centre (CNIO), Melchor Fernández Almagro 3, Madrid, E-28029, Spain.

^2^ Department of Ecological and Biological Sciences, Università degli Studi della Tuscia, Viterbo, Italy

^3^ Dipartimento di Biologia e Biotecnologie “C. Darwin”. Sapienza Università di Roma,

Rome, Italy

* *Correspondence:*

Giuseppe Bosso (gbosso@cnio.es) and Giovanni Cenci (gianni.cenci@uniroma1.it)

**Supplementary Figure Legends**

**Supplementary Figure 1. CBX3 gene is amplified in a plethora of human cancers and the concomitant gene amplification with either EGFR or RAC1 is not associated to any increase in genome instability. (A)** Chart showing the frequency of CBX3 gene amplification in different cancer types from TCGA Pan Cancer Atlas. (**B-E**) Charts showing that the simultaneous high-grade gene amplification of CBX3 with either (B, C) EGFR or (D, E) RAC1 is not associated to any increase in either genome aneuploidy (B, D) or in the fraction of genome altered (C, E). Data are expressed as mean ± SEM; n= samples per group. * P<0.05; ** P<0.01; *** P<0.001, ****P<0.0001, ns=not significant. One-way ANOVA (B-E).

**Supplementary Figure 2. CBX3 gene amplification is associated to low-grade and high-grade EGFR gene amplification**. (**A-I**) Charts showing that EGFR gene amplification is coupled to a low-grade and high-grade increase in CBX3 gene copy number in (A) esophagus and stomach cancers, (B) non-small cell lung cancer, (C) urothelial cancer, (D) endometrial cancer, (E) glioblastoma multiforme, (F) head and neck cancer, (G) breast cancer, (H) skin melanoma, (I) prostate cancer. n= number of samples.

**Supplementary Figure 3. CBX3 copy number variations are associated to a perturbation in CBX3 mRNA in human cancer. (A-J)** Charts showing that low-grade and high-grade copy number alterations in CBX3 gene are associated to significant changes in the levels of CBX3 transcripts in the (A) TCGA Pan Cancer Atlas, (B) esophagus and stomach cancers, (C) skin melanoma, (D) urothelial cancer, (E) head and neck cancer, (F) endometrial cancer, (G) breast cancer, (H) non-small cell lung cancer, (I) glioblastoma multiforme, (J) prostate cancer. Data are expressed as mean ± SEM; n= samples per group. * P<0.05; ** P<0.01; *** P<0.001, ****P<0.0001, ns=not significant. One-way ANOVA.

**Supplementary Figure 4. EGFR copy number variations are associated to a perturbation in EGFR mRNA in human cancer. (A-J)** Charts showing that high-grade amplification, and to a lesser extent low-grade CNVs, in EGFR gene are associated to significant changes in the levels of EGFR transcripts in the (A) TCGA Pan Cancer Atlas, (B) esophagus and stomach cancers, (C) skin melanoma, (D) urothelial cancer, (E) head and neck cancer, (F) endometrial cancer, (G) breast cancer, (H) non-small cell lung cancer, (I) glioblastoma multiforme, (J) prostate cancer. (**K**) Chart showing that the TCGA Pan Cancer Atlas specimens harboring CBX3 gene amplification display a significant increase in the transcriptional expression of EGFR.

Data are expressed as mean ± SEM; n= samples per group. * P<0.05; ** P<0.01; *** P<0.001, ****P<0.0001, ns=not significant. One-way ANOVA.

**Supplementary Figure 5. RAC1 gene amplification is associated to low-grade and high-grade CBX3 gene amplification**. (**A-I**) Charts showing that RAC1 gene amplification is coupled to a low-grade and high-grade increase in CBX3 gene copy number in (A) esophagus and stomach cancers, (B) non-small cell lung cancer, (C) urothelial cancer, (D) endometrial cancer, (E) glioblastoma multiforme, (F) head and neck cancer, (G) breast cancer, (H) skin melanoma, (I) prostate cancer. n= number of samples.

**Supplementary Figure 6. RAC1 copy number variations are associated to a perturbation in RAC1 mRNA in human cancer. (A-J)** Charts showing that low-grade and high-grade copy number alterations in RAC1 gene are associated to significant changes in the levels of RAC1 transcripts in the (A) TCGA Pan Cancer Atlas, (B) esophagus and stomach cancers, (C) skin melanoma, (D) urothelial cancer, (E) head and neck cancer, (F) endometrial cancer, (G) breast cancer, (H) non-small cell lung cancer, (I) glioblastoma multiforme, (J) prostate cancer. (**K**) Chart showing that the TCGA Pan Cancer Atlas specimens harboring RAC1 gene amplification display a significant increase in the transcriptional expression of CBX3. Data are expressed as mean ± SEM; n= samples per group. * P<0.05; ** P<0.01; *** P<0.001, ****P<0.0001, ns=not significant. One-way ANOVA.

**Supplementary Figure 7. CBX3 gene amplification negatively affects cancer patient lifespan. (A-C)** Survival curves showing the (A) overall survival, (B) disease-specific survival and (C) progression-free survival of TCGA Pan Cancer Atlas patients harboring high-grade amplification in CBX3 gene. n= samples per group. * P<0.05; ** P<0.01; *** P<0.001, ****P<0.0001, ns=not significant. Logrank test (A-C).

**Supplementary Figure 8. Simultaneous high expression of CBX3 and EGFR is associated to an increase in low- and high-grade amplification in CBX3 and EGFR genes.** (**A-C**) Charts showing the copy number status of CBX3 and EGFR genes in TCGA Pan Cancer Atlas specimens showing high transcriptional expression of (A) EGFR, (B) CBX3 and (C) both the genes. (**D-E**) Charts showing the enrichment in low-grade and high-grade gene amplification in TCGA Pan Cancer Atlas patients showing high simultaneous transcriptional expression of CBX3 and EGFR genes versus specimens showing high levels of only (D) CBX3 or (E) EGFR mRNAs. Z-score values higher than 1.5 were considered as being “high expression”. n=number of samples.

**Supplementary Figure 9. Low-grade copy number alterations in CBX3, EGFR and RAC1 genes co-occur in human cancer.** Charts (up) and contingency tables (bottom) respectively showing the overall copy number status and co-occurrence of shallow deletions as well as low-level gain in gene CN of CBX3 with either EGFR (**A**, **C**, **E**) or RAC1 (**B**, **D**, **F**) in glioblastoma multiforme (A, B), prostate cancer (C, D) and skin melanoma (E, F). n=number of samples. * P<0.05; ** P<0.01; *** P<0.001, ****P<0.0001, ns=not significant. Fisher’s exact test. GBM: glioblastoma multiforme.

**Supplementary Figure 10. Co-occurrence of low-grade copy number alterations in CBX3, EGFR and RAC1 genes is a frequent event in human cancer. (A-B)** Charts showing the frequency of concomitant gene copy number variations in CBX3 with either EGFR (A) or RAC1 (B) in the TCGA Pan Cancer Atlas cohort. (**C-F**) Contingency tables showing the co-occurrence of high-grade amplification of CBX3 gene with shallow deletions in (C) EGFR or (E) RAC1 genes as well as the co-occurrence of shallow deletions of CBX3 locus with low- and high-grade gene amplification in (D) EGFR or (F) RAC1 in the TCGA Pan Cancer Atlas patients. n=number of samples. * P<0.05; ** P<0.01; *** P<0.001, ****P<0.0001, ns=not significant. Fisher’s exact test.
