# Supplementary material for: Co-amplification of CBX3 with EGFR or RAC1 in human cancers corroborated by a conserved genetic interaction among the genes
Source: Cell Death Discov. 2023 Aug 26;9:317. doi: 10.1038/s41420-023-01598-5 (PMC10460438; doi:10.1038/s41420-023-01598-5)
Supplement: Supplementary file 2 — Supplementary Figure 1 [file 41420_2023_1598_MOESM2_ESM.pptx]

## Slide 1
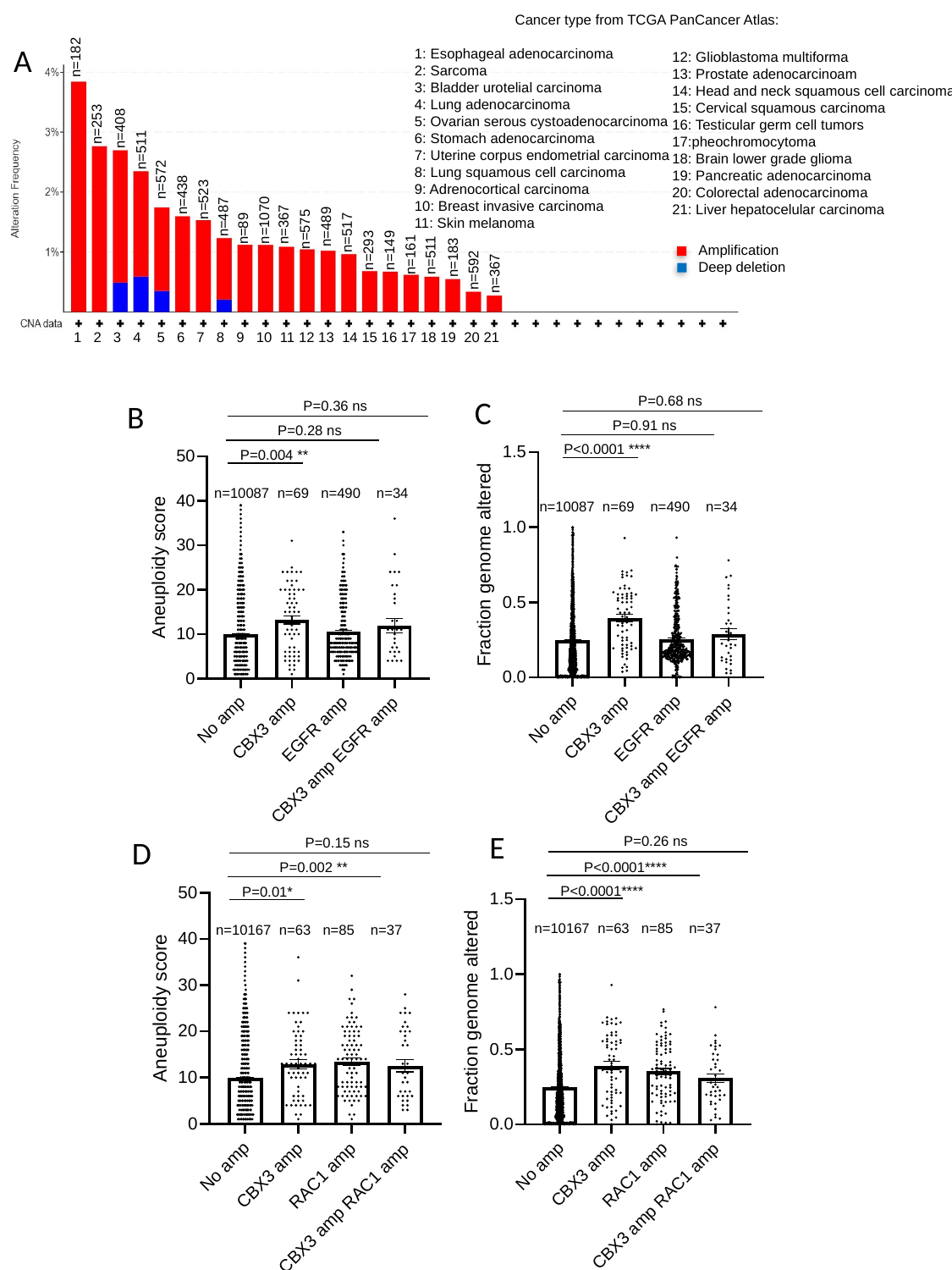

Cancer type from TCGA PanCancer Atlas:
1: Esophageal adenocarcinoma
2: Sarcoma
3: Bladder urotelial carcinoma
4: Lung adenocarcinoma
5: Ovarian serous cystoadenocarcinoma
6: Stomach adenocarcinoma
7: Uterine corpus endometrial carcinoma
8: Lung squamous cell carcinoma
9: Adrenocortical carcinoma
10: Breast invasive carcinoma
11: Skin melanoma
12: Glioblastoma multiforma
13: Prostate adenocarcinoam
14: Head and neck squamous cell carcinoma
15: Cervical squamous carcinoma
16: Testicular germ cell tumors
17:pheochromocytoma
18: Brain lower grade glioma
19: Pancreatic adenocarcinoma
20: Colorectal adenocarcinoma
21: Liver hepatocelular carcinoma
A
n=182
n=253
n=408
n=511
n=572
n=438
n=523
n=487
n=1070
n=367
n=89
n=489
n=575
n=517
n=293
n=149
n=161
n=511
Amplification
Deep deletion
n=183
n=592
n=367
1 2 3 4 5 6 7 8 9 10 11 12 13 14 15 16 17 18 19 20 21
P=0.68 ns
C
B
P=0.36 ns
P=0.91 ns
P=0.28 ns
P<0.0001 ****
P=0.004 **
n=10087 n=69 n=490 n=34
n=10087 n=69 n=490 n=34
E
P=0.26 ns
D
P=0.15 ns
P=0.002 **
P<0.0001****
P<0.0001****
P=0.01*
n=10167 n=63 n=85 n=37
n=10167 n=63 n=85 n=37
