# Supplementary material for: Co-amplification of CBX3 with EGFR or RAC1 in human cancers corroborated by a conserved genetic interaction among the genes
Source: Cell Death Discov. 2023 Aug 26;9:317. doi: 10.1038/s41420-023-01598-5 (PMC10460438; doi:10.1038/s41420-023-01598-5)
Supplement: Supplementary file 3 — Supplementary Figure 2 [file 41420_2023_1598_MOESM3_ESM.pptx]

## Slide 1
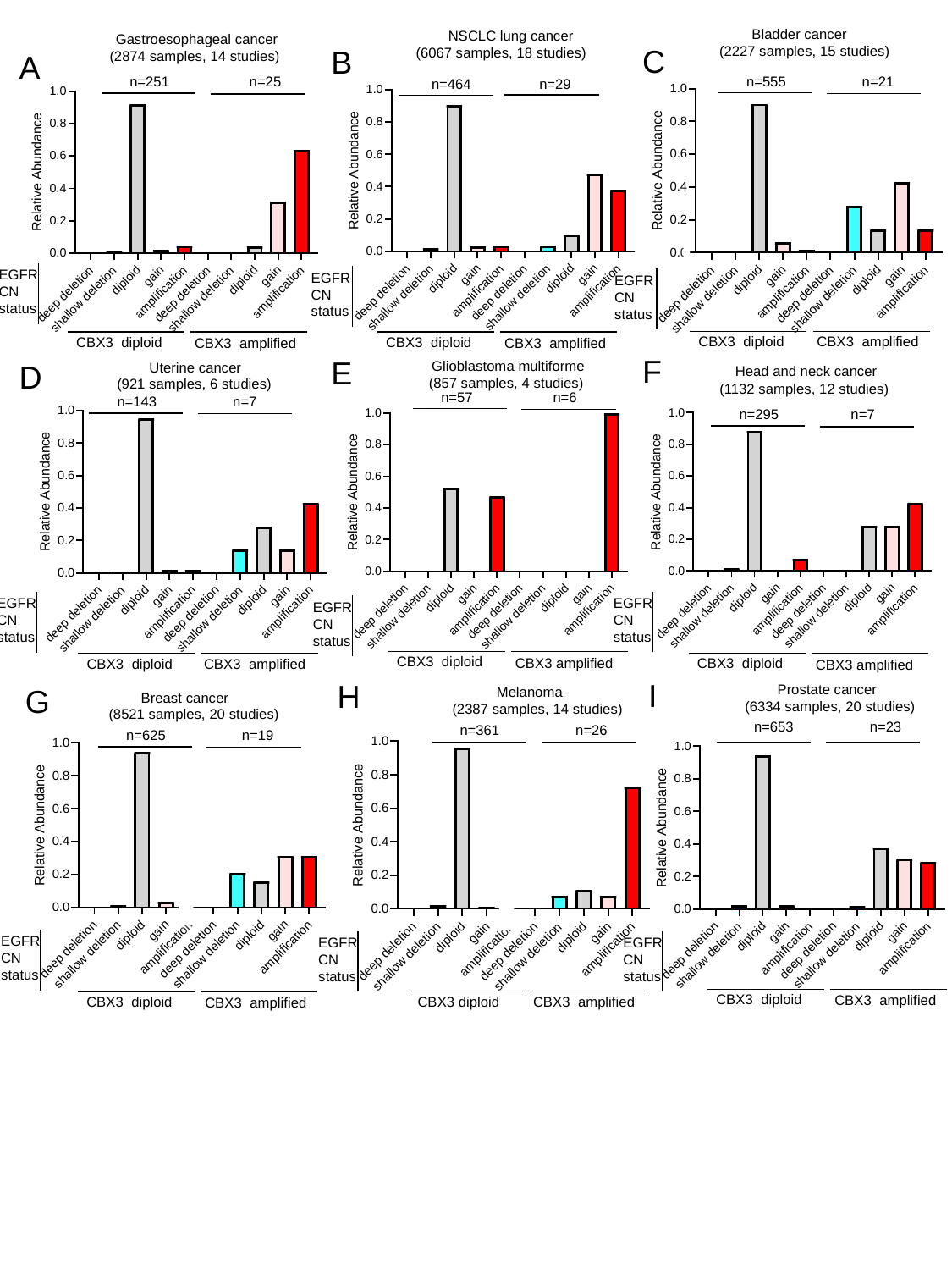

Bladder cancer
(2227 samples, 15 studies)
 NSCLC lung cancer
(6067 samples, 18 studies)
 Gastroesophageal cancer
 (2874 samples, 14 studies)
C
B
A
n=555 n=21
n=251 n=25
n=464 n=29
EGFR
CN
status
EGFR
CN
status
EGFR
CN
status
CBX3 diploid
CBX3 amplified
CBX3 diploid
CBX3 diploid
CBX3 amplified
CBX3 amplified
F
E
 Glioblastoma multiforme
 (857 samples, 4 studies)
D
 Uterine cancer
(921 samples, 6 studies)
 Head and neck cancer
 (1132 samples, 12 studies)
n=57 n=6
n=143 n=7
n=295 n=7
EGFR
CN
status
EGFR
CN
status
EGFR
CN
status
CBX3 diploid
CBX3 amplified
CBX3 diploid
CBX3 diploid
CBX3 amplified
CBX3 amplified
I
H
 Prostate cancer
(6334 samples, 20 studies)
G
 Melanoma
(2387 samples, 14 studies)
 Breast cancer
(8521 samples, 20 studies)
n=653 n=23
n=361 n=26
n=625 n=19
EGFR
CN
status
EGFR
CN
status
EGFR
CN
status
CBX3 diploid
CBX3 amplified
CBX3 diploid
CBX3 amplified
CBX3 diploid
CBX3 amplified
