# Supplementary material for: Co-amplification of CBX3 with EGFR or RAC1 in human cancers corroborated by a conserved genetic interaction among the genes
Source: Cell Death Discov. 2023 Aug 26;9:317. doi: 10.1038/s41420-023-01598-5 (PMC10460438; doi:10.1038/s41420-023-01598-5)
Supplement: Supplementary file 4 — Supplementary Figure 3 [file 41420_2023_1598_MOESM4_ESM.pptx]

## Slide 1
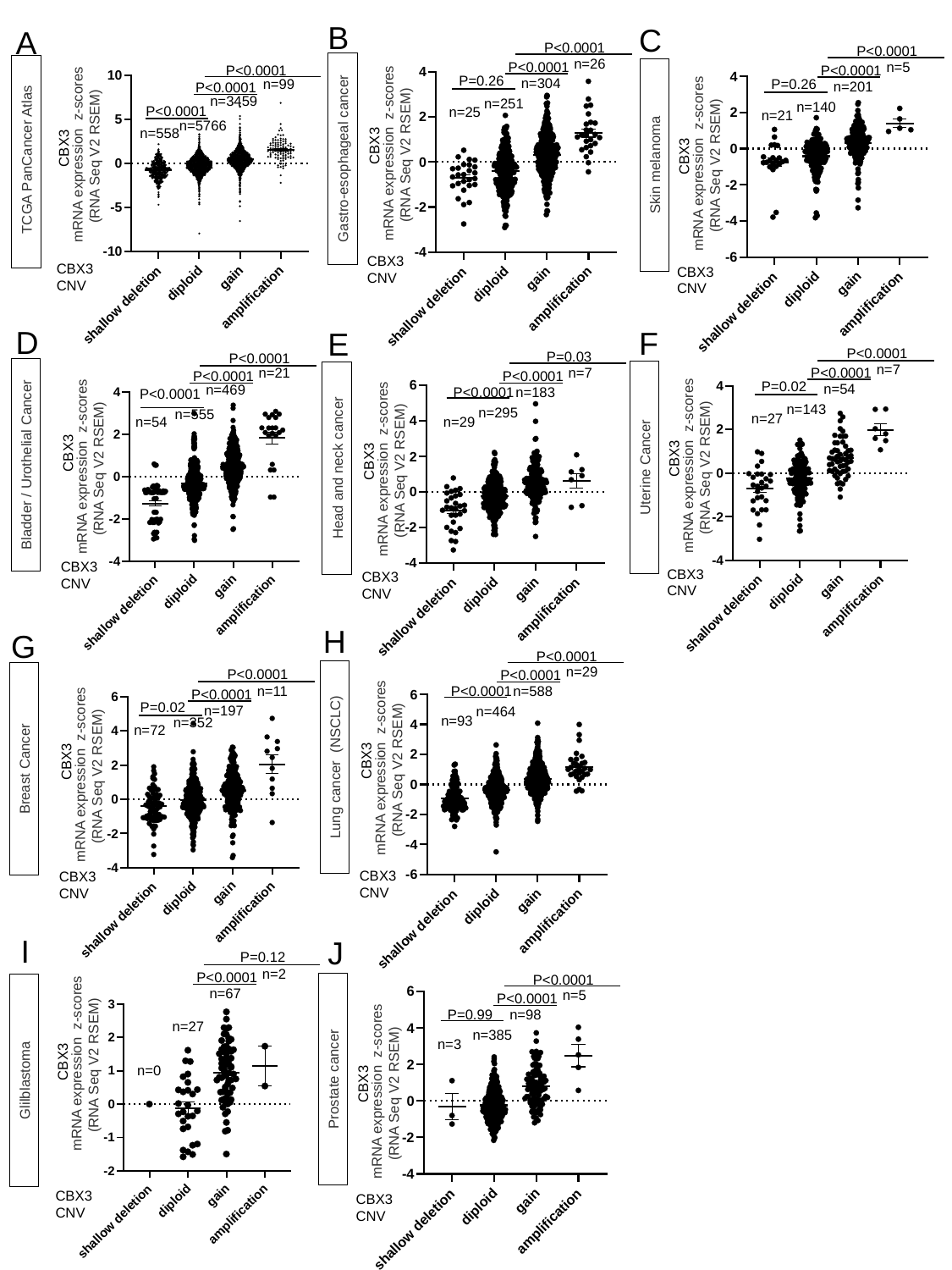

B
C
A
P<0.0001
P<0.0001
n=26
n=5
P<0.0001
P<0.0001
P<0.0001
P=0.26
n=304
P=0.26
n=99
n=201
P<0.0001
n=3459
n=251
n=140
P<0.0001
n=25
n=21
n=5766
n=558
CBX3
CBX3
 mRNA expression z-scores
(RNA Seq V2 RSEM)
 mRNA expression z-scores
(RNA Seq V2 RSEM)
CBX3
 mRNA expression z-scores
(RNA Seq V2 RSEM)
Gastro-esophageal cancer
 TCGA PanCancer Atlas
Skin melanoma
CBX3 CNV
CBX3 CNV
CBX3 CNV
D
F
E
P<0.0001
P=0.03
P<0.0001
n=7
P<0.0001
n=7
n=21
P<0.0001
P<0.0001
P=0.02
n=54
n=469
P<0.0001
n=183
P<0.0001
n=143
n=295
n=555
n=27
n=29
n=54
CBX3
CBX3
CBX3
 mRNA expression z-scores
(RNA Seq V2 RSEM)
 mRNA expression z-scores
(RNA Seq V2 RSEM)
 mRNA expression z-scores
(RNA Seq V2 RSEM)
Bladder / Urothelial Cancer
Uterine Cancer
Head and neck cancer
CBX3 CNV
CBX3 CNV
CBX3 CNV
H
G
P<0.0001
n=29
P<0.0001
P<0.0001
n=11
P<0.0001
n=588
P<0.0001
P=0.02
n=197
n=464
n=93
n=352
n=72
CBX3
CBX3
 mRNA expression z-scores
(RNA Seq V2 RSEM)
Lung cancer (NSCLC)
 mRNA expression z-scores
(RNA Seq V2 RSEM)
Breast Cancer
CBX3 CNV
CBX3 CNV
I
J
P=0.12
n=2
P<0.0001
P<0.0001
n=67
n=5
P<0.0001
P=0.99
n=98
n=27
n=385
n=3
CBX3
 mRNA expression z-scores
(RNA Seq V2 RSEM)
n=0
CBX3
Prostate cancer
Glilblastoma
 mRNA expression z-scores
(RNA Seq V2 RSEM)
CBX3 CNV
CBX3 CNV
