# Supplementary material for: Co-amplification of CBX3 with EGFR or RAC1 in human cancers corroborated by a conserved genetic interaction among the genes
Source: Cell Death Discov. 2023 Aug 26;9:317. doi: 10.1038/s41420-023-01598-5 (PMC10460438; doi:10.1038/s41420-023-01598-5)
Supplement: Supplementary file 5 — Supplementary Figure 4 [file 41420_2023_1598_MOESM5_ESM.pptx]

## Slide 1
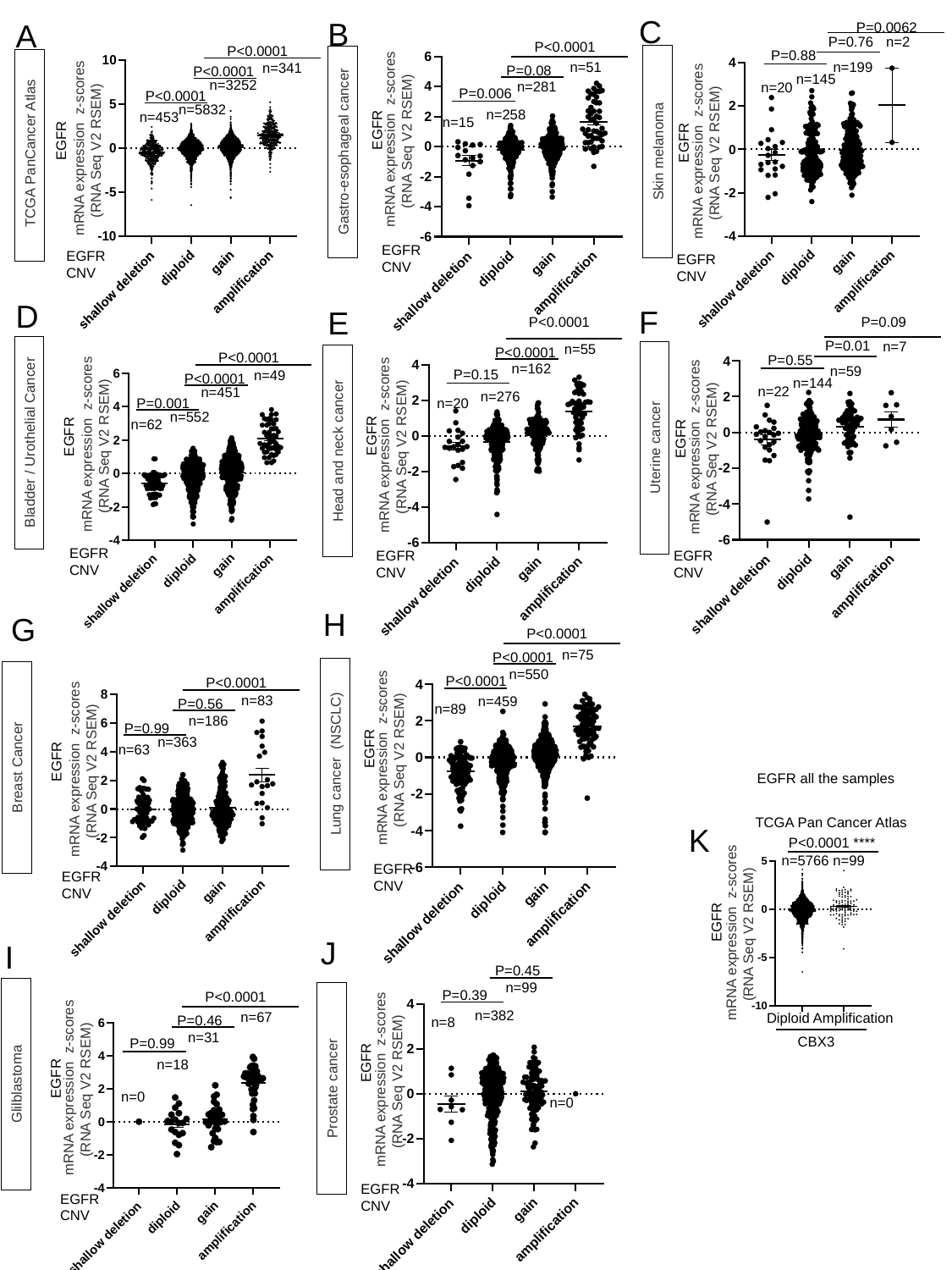

C
B
A
P=0.0062
P=0.76
n=2
P<0.0001
P<0.0001
P=0.88
n=51
n=199
n=341
P=0.08
P<0.0001
n=145
n=3252
n=281
n=20
P=0.006
P<0.0001
n=5832
n=258
n=453
n=15
EGFR
 mRNA expression z-scores
(RNA Seq V2 RSEM)
EGFR
EGFR
 mRNA expression z-scores
(RNA Seq V2 RSEM)
 mRNA expression z-scores
(RNA Seq V2 RSEM)
Skin melanoma
Gastro-esophageal cancer
 TCGA PanCancer Atlas
EGFRCNV
EGFRCNV
EGFR CNV
D
F
E
P<0.0001
P=0.09
P=0.01
n=7
n=55
P<0.0001
P<0.0001
P=0.55
n=162
n=59
P=0.15
n=49
P<0.0001
n=144
n=22
n=451
n=276
P=0.001
n=20
n=552
n=62
EGFR
EGFR
EGFR
 mRNA expression z-scores
(RNA Seq V2 RSEM)
 mRNA expression z-scores
(RNA Seq V2 RSEM)
 mRNA expression z-scores
(RNA Seq V2 RSEM)
Bladder / Urothelial Cancer
Uterine cancer
Head and neck cancer
EGFRCNV
EGFR CNV
EGFRCNV
H
G
P<0.0001
n=75
P<0.0001
n=550
P<0.0001
P<0.0001
n=83
n=459
P=0.56
n=89
n=186
P=0.99
n=363
EGFR
n=63
 mRNA expression z-scores
(RNA Seq V2 RSEM)
EGFR
 mRNA expression z-scores
(RNA Seq V2 RSEM)
Lung cancer (NSCLC)
Breast Cancer
EGFR all the samples
TCGA Pan Cancer Atlas
K
P<0.0001 ****
n=5766 n=99
EGFRCNV
EGFRCNV
EGFR
 mRNA expression z-scores
(RNA Seq V2 RSEM)
J
I
P=0.45
n=99
P=0.39
P<0.0001
n=382
n=67
Diploid Amplification
P=0.46
n=8
n=31
CBX3
P=0.99
EGFR
n=18
 mRNA expression z-scores
(RNA Seq V2 RSEM)
EGFR
 mRNA expression z-scores
(RNA Seq V2 RSEM)
Glilblastoma
Prostate cancer
n=0
n=0
EGFRCNV
EGFRCNV
