# Supplementary material for: Co-amplification of CBX3 with EGFR or RAC1 in human cancers corroborated by a conserved genetic interaction among the genes
Source: Cell Death Discov. 2023 Aug 26;9:317. doi: 10.1038/s41420-023-01598-5 (PMC10460438; doi:10.1038/s41420-023-01598-5)
Supplement: Supplementary file 6 — Supplementary Figure 5 [file 41420_2023_1598_MOESM6_ESM.pptx]

## Slide 1
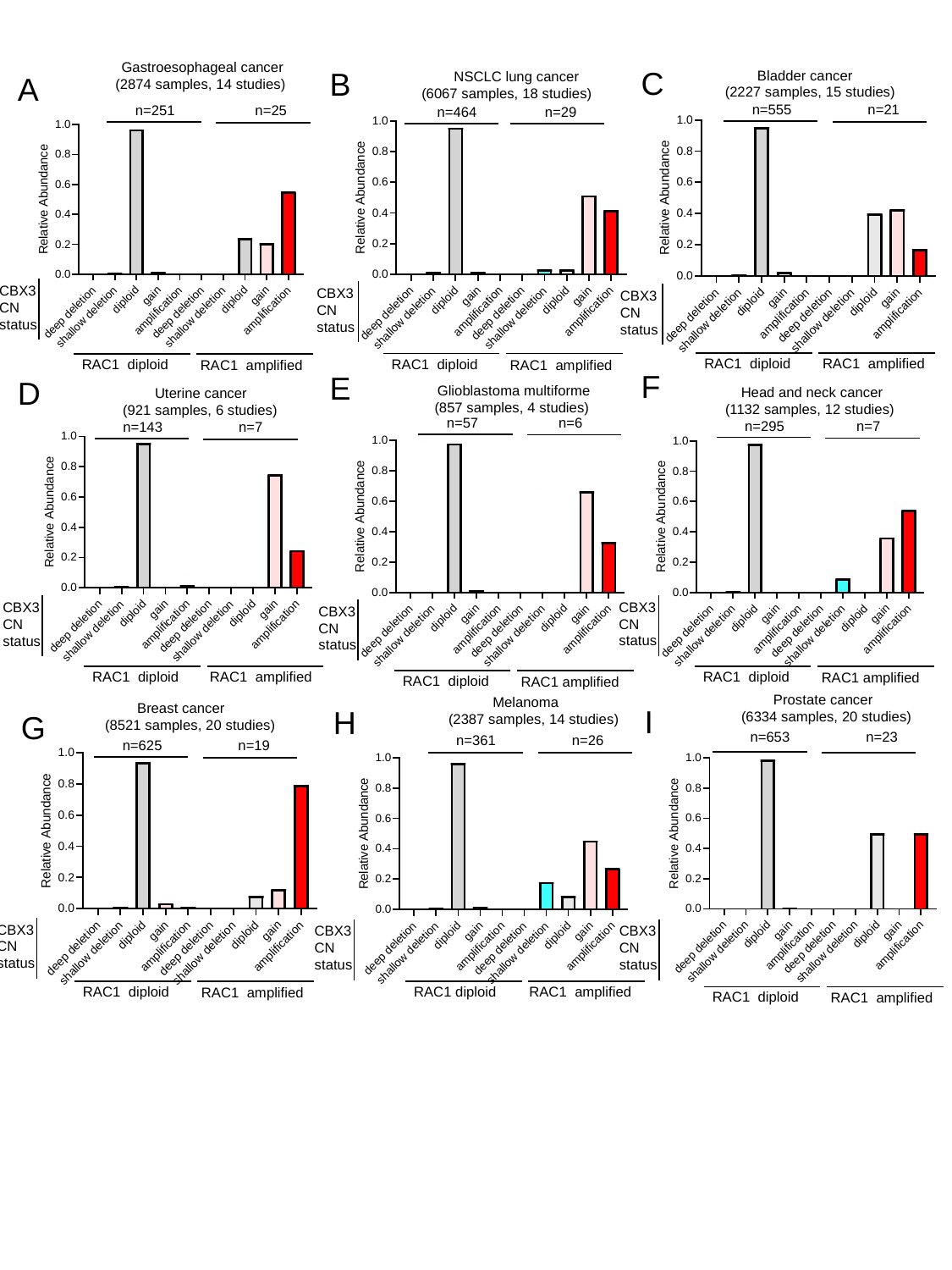

Gastroesophageal cancer
 (2874 samples, 14 studies)
C
B
 Bladder cancer
(2227 samples, 15 studies)
 NSCLC lung cancer
(6067 samples, 18 studies)
A
n=555 n=21
n=251 n=25
n=464 n=29
CBX3
CN
status
CBX3
CN
status
CBX3
CN
status
RAC1 diploid
RAC1 amplified
RAC1 diploid
RAC1 diploid
RAC1 amplified
RAC1 amplified
F
E
D
 Glioblastoma multiforme
 (857 samples, 4 studies)
 Head and neck cancer
 (1132 samples, 12 studies)
 Uterine cancer
(921 samples, 6 studies)
n=57 n=6
n=295 n=7
n=143 n=7
CBX3
CN
status
CBX3
CN
status
CBX3
CN
status
RAC1 diploid
RAC1 diploid
RAC1 amplified
RAC1 amplified
RAC1 diploid
RAC1 amplified
 Prostate cancer
(6334 samples, 20 studies)
 Melanoma
(2387 samples, 14 studies)
 Breast cancer
(8521 samples, 20 studies)
I
H
G
n=653 n=23
n=361 n=26
n=625 n=19
CBX3
CN
status
CBX3
CN
status
CBX3
CN
status
RAC1 diploid
RAC1 amplified
RAC1 diploid
RAC1 amplified
RAC1 diploid
RAC1 amplified
