# Supplementary material for: Co-amplification of CBX3 with EGFR or RAC1 in human cancers corroborated by a conserved genetic interaction among the genes
Source: Cell Death Discov. 2023 Aug 26;9:317. doi: 10.1038/s41420-023-01598-5 (PMC10460438; doi:10.1038/s41420-023-01598-5)
Supplement: Supplementary file 7 — Supplementary Figure 6 [file 41420_2023_1598_MOESM7_ESM.pptx]

## Slide 1
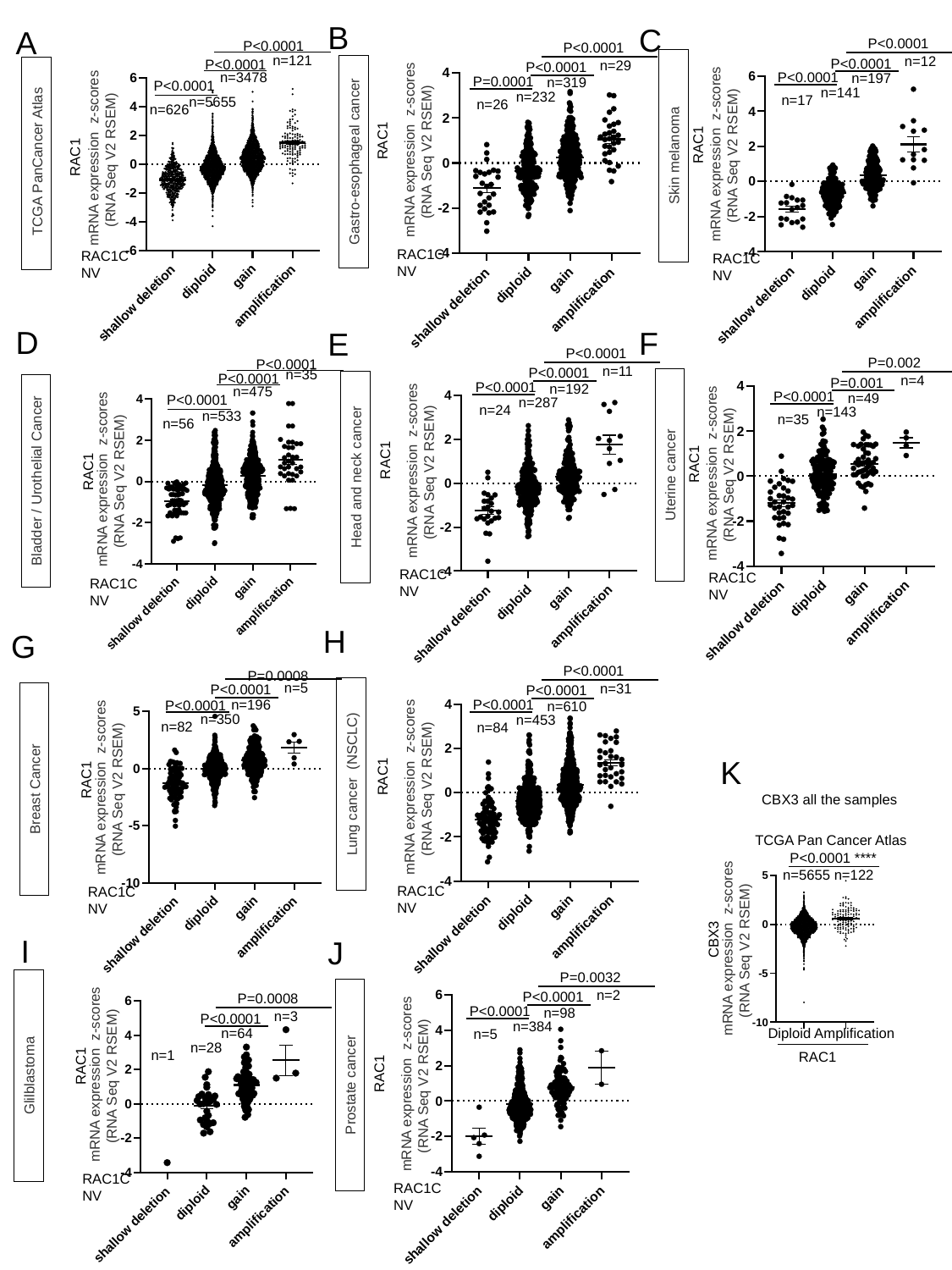

B
C
A
P<0.0001
P<0.0001
P<0.0001
n=121
n=12
P<0.0001
P<0.0001
n=29
P<0.0001
n=3478
P<0.0001
n=197
P=0.0001
n=319
P<0.0001
n=141
n=232
n=17
n=5655
n=26
n=626
RAC1
RAC1
 mRNA expression z-scores
(RNA Seq V2 RSEM)
 mRNA expression z-scores
(RNA Seq V2 RSEM)
 mRNA expression z-scores
(RNA Seq V2 RSEM)
RAC1
Skin melanoma
Gastro-esophageal cancer
 TCGA PanCancer Atlas
RAC1CNV
RAC1CNV
RAC1CNV
D
F
E
P<0.0001
P=0.002
P<0.0001
n=11
P<0.0001
n=35
P<0.0001
n=4
P=0.001
P<0.0001
n=192
n=475
P<0.0001
n=49
P<0.0001
n=287
n=24
n=143
n=533
n=35
n=56
RAC1
RAC1
 mRNA expression z-scores
(RNA Seq V2 RSEM)
RAC1
 mRNA expression z-scores
(RNA Seq V2 RSEM)
 mRNA expression z-scores
(RNA Seq V2 RSEM)
Uterine cancer
Head and neck cancer
Bladder / Urothelial Cancer
RAC1CNV
RAC1CNV
RAC1CNV
H
G
P<0.0001
P=0.0008
n=5
n=31
P<0.0001
P<0.0001
n=196
P<0.0001
P<0.0001
n=610
n=350
n=453
n=82
n=84
K
RAC1
RAC1
 mRNA expression z-scores
(RNA Seq V2 RSEM)
 mRNA expression z-scores
(RNA Seq V2 RSEM)
Lung cancer (NSCLC)
Breast Cancer
CBX3 all the samples
TCGA Pan Cancer Atlas
P<0.0001 ****
n=5655 n=122
RAC1CNV
RAC1CNV
CBX3
I
J
 mRNA expression z-scores
(RNA Seq V2 RSEM)
P=0.0032
n=2
P<0.0001
P=0.0008
P<0.0001
n=98
n=3
P<0.0001
n=384
Diploid Amplification
n=64
n=5
n=28
n=1
RAC1
RAC1
 mRNA expression z-scores
(RNA Seq V2 RSEM)
RAC1
Glilblastoma
 mRNA expression z-scores
(RNA Seq V2 RSEM)
Prostate cancer
RAC1CNV
RAC1CNV
