# Supplementary material for: Co-amplification of CBX3 with EGFR or RAC1 in human cancers corroborated by a conserved genetic interaction among the genes
Source: Cell Death Discov. 2023 Aug 26;9:317. doi: 10.1038/s41420-023-01598-5 (PMC10460438; doi:10.1038/s41420-023-01598-5)
Supplement: Supplementary file 8 — Supplementary Figure 7 [file 41420_2023_1598_MOESM8_ESM.pptx]

## Slide 1
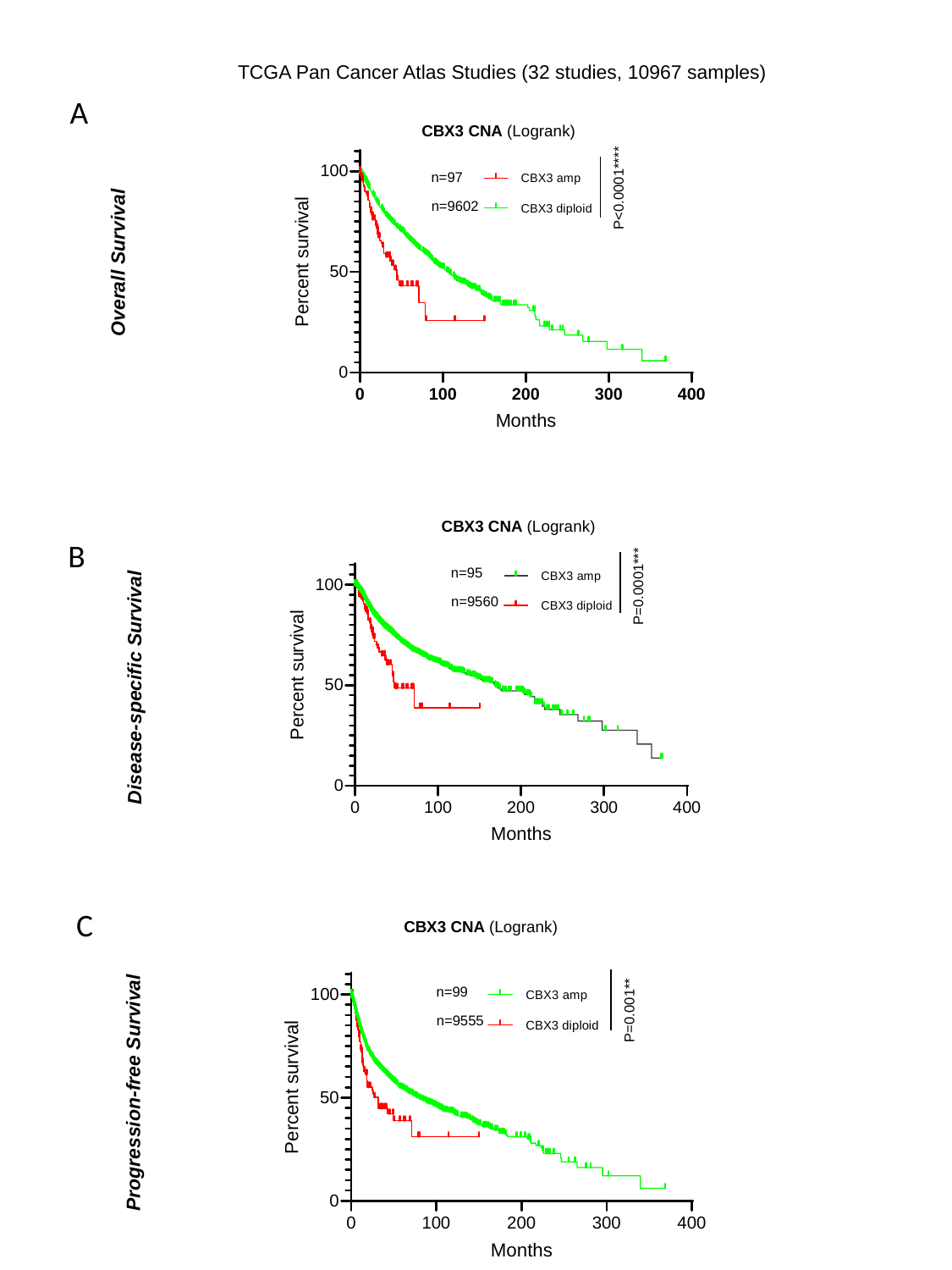

TCGA Pan Cancer Atlas Studies (32 studies, 10967 samples)
A
CBX3 CNA (Logrank)
n=97
P<0.0001****
n=9602
Overall Survival
CBX3 CNA (Logrank)
B
n=95
P=0.0001***
n=9560
Disease-specific Survival
C
CBX3 CNA (Logrank)
n=99
P=0.001**
n=9555
Progression-free Survival
