# Supplementary material for: Co-amplification of CBX3 with EGFR or RAC1 in human cancers corroborated by a conserved genetic interaction among the genes
Source: Cell Death Discov. 2023 Aug 26;9:317. doi: 10.1038/s41420-023-01598-5 (PMC10460438; doi:10.1038/s41420-023-01598-5)
Supplement: Supplementary file 9 — Supplementary Figure 8 [file 41420_2023_1598_MOESM9_ESM.pptx]

## Slide 1
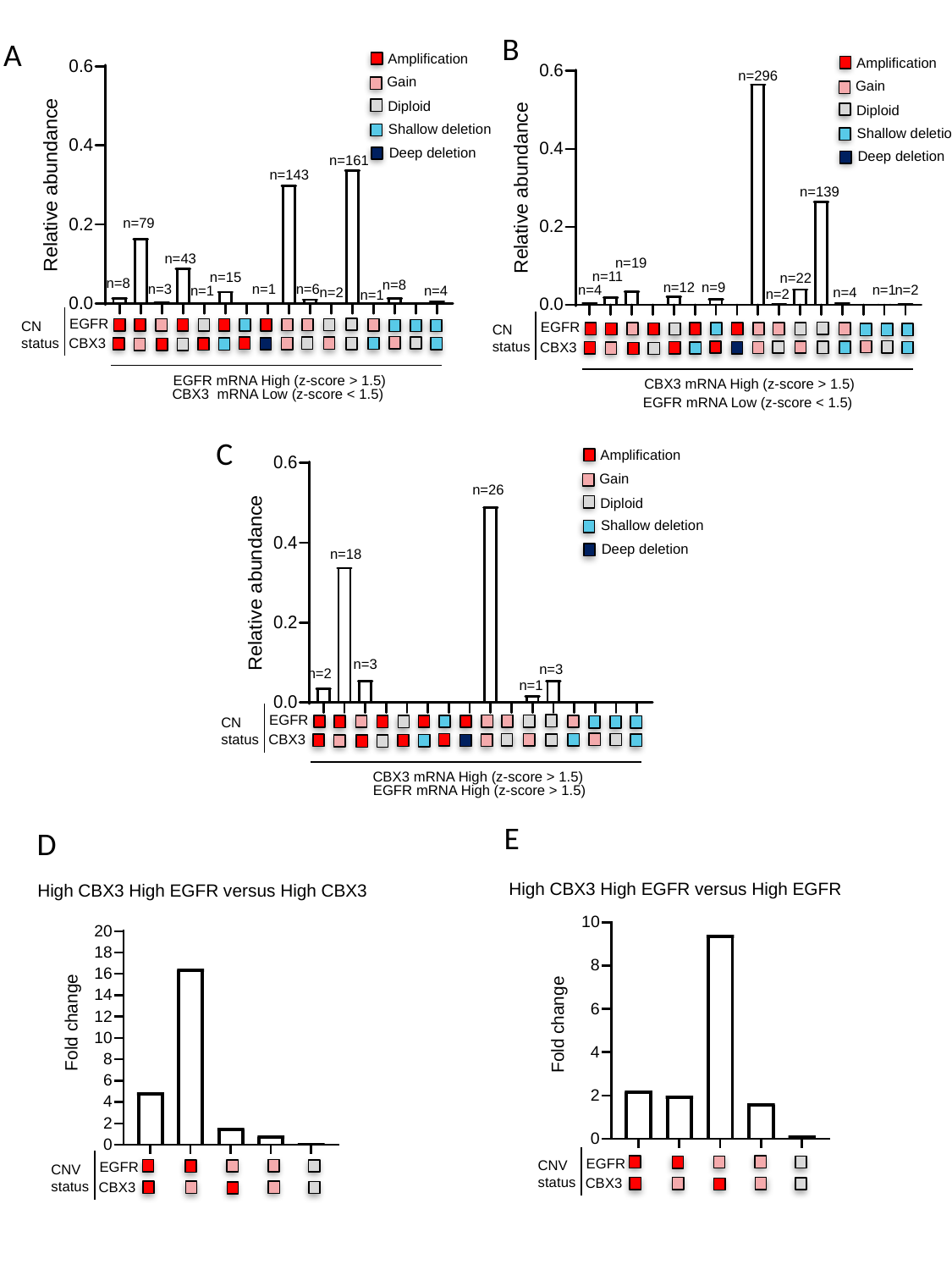

B
A
Amplification
Amplification
 n=296
Gain
Gain
Diploid
Diploid
Shallow deletion
Shallow deletion
Deep deletion
Deep deletion
 n=161
 n=143
 n=139
 n=79
 n=43
 n=19
 n=11
 n=15
 n=22
 n=8
 n=8
 n=9
 n=12
 n=6
 n=1
 n=3
 n=4
 n=2
 n=1
 n=1
 n=4
 n=2
 n=4
 n=2
 n=1
EGFR
CN status
EGFR
CN status
CBX3
CBX3
EGFR mRNA High (z-score > 1.5)
CBX3 mRNA High (z-score > 1.5)
CBX3 mRNA Low (z-score < 1.5)
EGFR mRNA Low (z-score < 1.5)
C
Amplification
Gain
n=26
Diploid
Shallow deletion
Deep deletion
n=18
n=3
n=3
n=2
n=1
EGFR
CN status
CBX3
CBX3 mRNA High (z-score > 1.5)
EGFR mRNA High (z-score > 1.5)
E
D
High CBX3 High EGFR versus High EGFR
High CBX3 High EGFR versus High CBX3
Fold change
Fold change
EGFR
CNV status
EGFR
CNV status
CBX3
CBX3
