# Supplementary material for: Co-amplification of CBX3 with EGFR or RAC1 in human cancers corroborated by a conserved genetic interaction among the genes
Source: Cell Death Discov. 2023 Aug 26;9:317. doi: 10.1038/s41420-023-01598-5 (PMC10460438; doi:10.1038/s41420-023-01598-5)
Supplement: Supplementary file 10 — Supplementary Figure 9 [file 41420_2023_1598_MOESM10_ESM.pptx]

## Slide 1
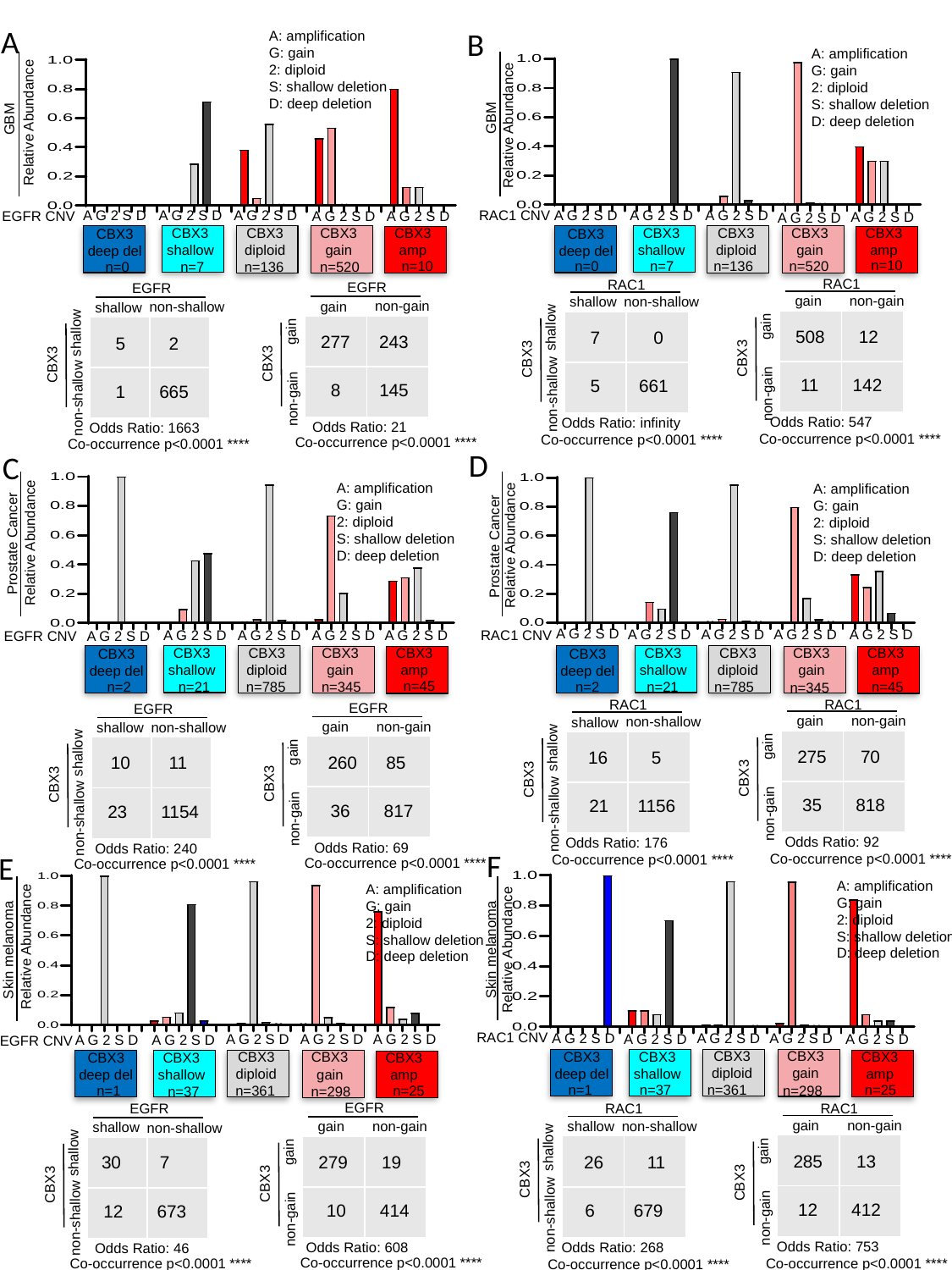

A
B
A: amplification
G: gain
2: diploid
S: shallow deletion D: deep deletion
A: amplification
G: gain
2: diploid
S: shallow deletion D: deep deletion
GBM
GBM
Relative Abundance
Relative Abundance
A G 2 S D
A G 2 S D
RAC1 CNV
A G 2 S D
A G 2 S D
A G 2 S D
A G 2 S D
A G 2 S D
A G 2 S D
EGFR CNV
A G 2 S D
A G 2 S D
CBX3
diploid
CBX3
diploid
CBX3
gain
CBX3
gain
CBX3
shallow
CBX3
shallow
CBX3
amp
CBX3
amp
CBX3
deep del
CBX3
deep del
n=10
n=0
n=136
n=7
n=10
n=520
n=0
n=136
n=7
n=520
RAC1
RAC1
EGFR
EGFR
non-gain
gain
non-shallow
shallow
non-gain
gain
non-shallow
shallow
| | |
| --- | --- |
| | |
| | |
| --- | --- |
| | |
gain
shallow
| | |
| --- | --- |
| | |
| | |
| --- | --- |
| | |
gain
shallow
508 12
 7 0
277 243
 5 2
CBX3
CBX3
CBX3
CBX3
 11 142
 5 661
 8 145
 1 665
non-gain
non-shallow
non-shallow
non-gain
Odds Ratio: 547
Odds Ratio: infinity
Odds Ratio: 21
Odds Ratio: 1663
Co-occurrence p<0.0001 ****
Co-occurrence p<0.0001 ****
Co-occurrence p<0.0001 ****
Co-occurrence p<0.0001 ****
D
C
A: amplification
G: gain
2: diploid
S: shallow deletion D: deep deletion
A: amplification
G: gain
2: diploid
S: shallow deletion D: deep deletion
Prostate Cancer
Prostate Cancer
Relative Abundance
Relative Abundance
A G 2 S D
A G 2 S D
A G 2 S D
A G 2 S D
A G 2 S D
A G 2 S D
A G 2 S D
A G 2 S D
RAC1 CNV
A G 2 S D
A G 2 S D
EGFR CNV
CBX3
diploid
CBX3
diploid
CBX3
gain
CBX3
gain
CBX3
shallow
CBX3
shallow
CBX3
amp
CBX3
amp
CBX3
deep del
CBX3
deep del
n=45
n=45
n=2
n=785
n=2
n=785
n=21
n=21
n=345
n=345
RAC1
RAC1
EGFR
EGFR
non-gain
gain
non-shallow
shallow
gain
non-gain
shallow
non-shallow
| | |
| --- | --- |
| | |
| | |
| --- | --- |
| | |
gain
shallow
| | |
| --- | --- |
| | |
| | |
| --- | --- |
| | |
gain
shallow
275 70
 16 5
260 85
 10 11
CBX3
CBX3
CBX3
CBX3
 35 818
 21 1156
 36 817
 23 1154
non-gain
non-shallow
non-shallow
non-gain
Odds Ratio: 92
Odds Ratio: 176
Odds Ratio: 69
Odds Ratio: 240
F
E
Co-occurrence p<0.0001 ****
Co-occurrence p<0.0001 ****
Co-occurrence p<0.0001 ****
Co-occurrence p<0.0001 ****
A: amplification
G: gain
2: diploid
S: shallow deletion D: deep deletion
A: amplification
G: gain
2: diploid
S: shallow deletion D: deep deletion
Skin melanoma
Skin melanoma
Relative Abundance
Relative Abundance
RAC1 CNV
A G 2 S D
A G 2 S D
A G 2 S D
A G 2 S D
A G 2 S D
A G 2 S D
A G 2 S D
A G 2 S D
A G 2 S D
A G 2 S D
EGFR CNV
CBX3
diploid
CBX3
gain
CBX3
shallow
CBX3
amp
CBX3
deep del
CBX3
diploid
CBX3
gain
CBX3
shallow
CBX3
amp
CBX3
deep del
n=25
n=1
n=361
n=37
n=298
n=25
n=1
n=361
n=37
n=298
EGFR
RAC1
EGFR
RAC1
non-gain
gain
non-shallow
non-gain
shallow
gain
shallow
non-shallow
shallow
| | |
| --- | --- |
| | |
| | |
| --- | --- |
| | |
| | |
| --- | --- |
| | |
gain
| | |
| --- | --- |
| | |
gain
shallow
285 13
 26 11
279 19
 30 7
CBX3
CBX3
CBX3
CBX3
 12 412
 6 679
 10 414
 12 673
non-shallow
non-gain
non-shallow
non-gain
Odds Ratio: 753
Odds Ratio: 268
Odds Ratio: 608
Odds Ratio: 46
Co-occurrence p<0.0001 ****
Co-occurrence p<0.0001 ****
Co-occurrence p<0.0001 ****
Co-occurrence p<0.0001 ****
