# Supplementary material for: Co-amplification of CBX3 with EGFR or RAC1 in human cancers corroborated by a conserved genetic interaction among the genes
Source: Cell Death Discov. 2023 Aug 26;9:317. doi: 10.1038/s41420-023-01598-5 (PMC10460438; doi:10.1038/s41420-023-01598-5)
Supplement: Supplementary file 11 — Supplementary Figure 10 [file 41420_2023_1598_MOESM11_ESM.pptx]

## Slide 1
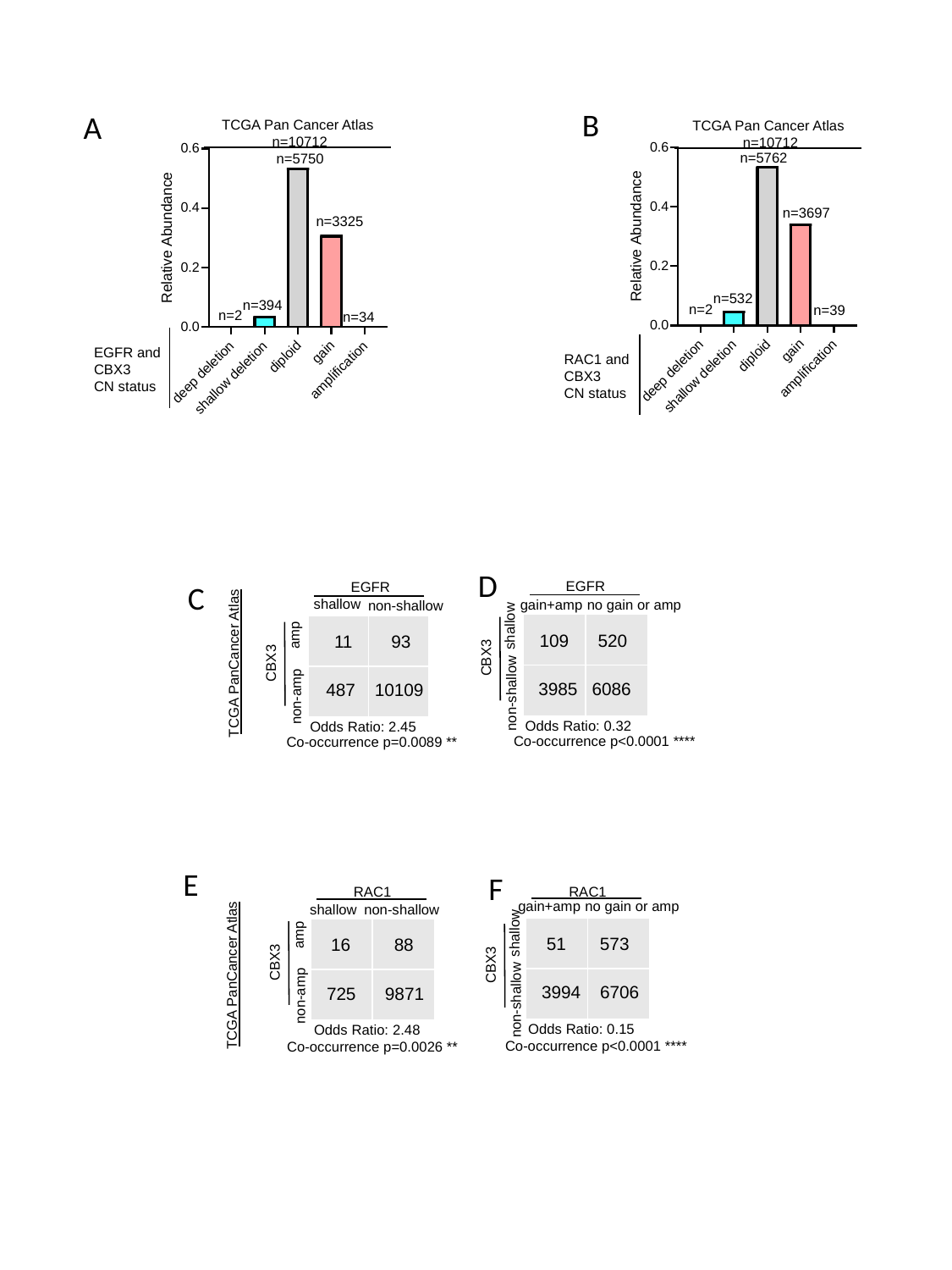

B
A
TCGA Pan Cancer Atlas
 n=10712
TCGA Pan Cancer Atlas
 n=10712
n=5762
n=5750
n=3697
n=3325
n=532
n=394
n=2
n=39
n=2
n=34
EGFR and
CBX3
CN status
RAC1 and
CBX3
CN status
D
EGFR
EGFR
C
shallow
no gain or amp
gain+amp
non-shallow
shallow
| | |
| --- | --- |
| | |
| | |
| --- | --- |
| | |
amp
109 520
 11 93
TCGA PanCancer Atlas
CBX3
CBX3
 3985 6086
 487 10109
non-shallow
non-amp
Odds Ratio: 0.32
Odds Ratio: 2.45
Co-occurrence p<0.0001 ****
Co-occurrence p=0.0089 **
E
F
RAC1
RAC1
no gain or amp
gain+amp
non-shallow
shallow
| | |
| --- | --- |
| | |
shallow
| | |
| --- | --- |
| | |
amp
 51 573
 16 88
CBX3
TCGA PanCancer Atlas
CBX3
3994 6706
725 9871
non-amp
non-shallow
Odds Ratio: 0.15
Odds Ratio: 2.48
Co-occurrence p<0.0001 ****
Co-occurrence p=0.0026 **
