# Supplementary material for: Co-amplification of CBX3 with EGFR or RAC1 in human cancers corroborated by a conserved genetic interaction among the genes
Source: Cell Death Discov. 2023 Aug 26;9:317. doi: 10.1038/s41420-023-01598-5 (PMC10460438; doi:10.1038/s41420-023-01598-5)
Supplement: Supplementary file 12 — Cancer Studies List [file 41420_2023_1598_MOESM12_ESM.docx]

**Cancer Studies List**

**Bladder / Upper tract urothelial cancer:**

Bladder Cancer (MSK/TCGA, 2020)

Bladder Cancer (MSKCC, Eur Urol 2014)

Bladder Cancer (MSKCC, J Clin Onco 2013)

Bladder Cancer (MSKCC, Nat Genet 2016)

Bladder Cancer (TCGA, Cell 2017)

Bladder Urothelial Carcinoma (BGI, Nat Genet 2013)

Bladder Urothelial Carcinoma (DFCI/MSKCC, Cancer Discov 2014)

Bladder Urothelial Carcinoma (TCGA, Firehose Legacy)

Non-muscle invasive Bladder Cancer (MSK Eur Urol 2017)

Urothelial Carcinoma (Cornell/Trento, Nat Gen 2016)

Upper tract urothelial cancer (MSK, Eur Urol 2015)

Upper tract urothelial carcinoma (Cornell/Baylor/MDACC, Nat Comm)

Upper tract urothelial carcinoma (IGBMC, Genome Biology 2021)

Upper tract urothelial carcinoma (MSK, Nat Commun 2020)

Upper tract urothelial carcinoma PDX (MSK, Nat Commun 2020)

**Breast Cancer:**

Breast cancer (MSK, Cancer cell 2018)

Breast cancer (MSK, Nature Cancer 2020)

Metastatic Breast Cancer (MSK, Cancer discovery 2021)

Breast fibroepithelial tumors (Duke-NUS, Nat Genet 2015)

Breast cancer (CPTAC, Cell 2020)

Breast Cancer (METABRIC, Nature 20212 & Nat Commun 2016)

Breast cancer (MSK, Clinical Cancer Res 2020)

Breast Cancer (MSKCC, NPJ Breast Cancer 2019)

Breast Cancer (SMC 2018)

Breast Cancer Xenografts (British Columbia, Nature 2015)

Breast Invasive Carcinoma (British Columbia, Nature 2012)

Breast invasive Carcinoma (Broad, Nature 2012)

Breast Invasive Carcinoma (Sanger,Nature 2012)

Breast Invasive Carcinoma (TCGA, Firehose Legacy)

Juvenile Papillomatosis and Breast Cancer (MSK, 2020)

MAPK on resistance to anti-HER2 therapy for breast cancer (MSKCC)

Metastatic breast cancer (INSERM, Plos Med 2016)

The metastatic breast cancer project (Provisional, February 2020)

Adenoid cystic carcinoma of the breast (MSKCC, J Pathol. 2015)

Metaplasic breast cancer (MSK, 2021)

**Brain tumor:**

Pediatric brain cancer (CPTAC/CHOP, Cell 2020)

Brain lower grade glioma (TCGA, PanCancer Atlas)

Diffuse glioma (GLASS Consortium Nature 2019)

Glioma (MSK, Nature 2019)

Glioma (MSKCC, Clin Cancer Res 2019)

Low-grade gliomas (UCSF, Science 2014)

Merged cohort of LGG and GBM (TCGA, Cell 2016)

Brain tumor PDXs (Mayo Clinic, 2019)

Glioblastoma (CPTAC, Cell 2021)

Glioblastoma (Columbia, Nat Med 2019)

Glioblastoma multiforme (TCGA, Firehose legacy)

Anaplastic Oligodendroglioma and anaplastic oligoastrocytoma (MSK)

Medulloblastoma (Broad Nature 2012)

Medulloblastoma (DKFZ, Nature 2017)

Medulloblastoma (ICGC, Nature 2012)

Medulloblastoma (PCGP, Nature 2012)

Medulloblastoma (Sickkids, Nature 2016)

Pilocytic Astrcytoma (ICGC, Nature Genetics 2013)

**Esophagus/Stomach Cancer:**

Esophageal/Stomach Cancer (MSK, 2020)

Esophageal squamous cell carcinoma (ICGC, Nature 2014)

Esophageal squamous cell carcinoma (UCLA, Nat Genet 2014)

Esophageal cancer-TRAP project (MSK, Lancet Oncol 2020)

Esophageal Carcinoma (TCGA, Nature 2017)

Gastric adenocarcinoma (TMUCIH, PNAS 2015)

Metastatic esophagogastric cancer (MSKCC, Cancer discovery 2017)

Esophageal adenocarcinoma (DFCI, Nat Genet 2013)

Esophageal Carcinoma (TCGA, Firehose legacy)

Gastric cancer (OncoSG, 2018)

Stomach adenocarcinoma (Pfizer and UHK, Nat Genet 2014)

Stomach adenocarcinoma (TCGA, Firehose legacy)

Stomach adenocarcinoma (U Tokyo, Nat Genet 2014)

Stomach adenocarcinoma (UHK, Nat Genet 2011)

**Head and neck cancer**:

Recurrent and metastatic head & neck cancer (MSKCC, JAMA Oncol)

Head and neck squamous cell carcinoma (John Hopkins, Science)

Head and neck squamous cell carcinoma (TCGA, Firehose Legacy)

Head and neck squamous cell carcinoma (Broad, Science 2011)

Oral squamous cell carcinoma (;D Anderson, Cancer discov 2013)

Nasopharyngeal carcinoma (Singapore, Nat Genet 2014)

Adenoid cystic carcinoma (FMI, AM J Surg Pathl. 2014)

Adenoid cystic carcinoma (JHU, Cancer Prev Res 2016)

Adenoid cystic carcinoma (MDA, Clin cancer re 2015)

Adenoid cystic carcinoma (MGH, Nat Gen 2016)

Adenoid cystic carcinoma (MSKCC, Nat Genet 2013)

Adenoid cystic carcinoma (Sanger/MDA, JCI 2013)

**Non-small cell lung cancer**:

Non-small cell lung cancer (MSK, Cancer cell 2018)

Non-small cell lung cancer (MSKCC, J ClinOncol 2018)

Non-small cell lung cancer (TRACERx, NEJM & Nature 2017)

Non-small cell lung cancer (University of Turin, Lung Cancer 2017)

Non-small cell lung cancer (MSK, Science 2015)

Pan-lung cancer (TCGA, Nat Genet 2016)

Lung Adenocarcinoma (Broad, Cell 2012)

Lung Adenocarcinoma (CPTAC, Cell 2020)

Lung Adenocarcinoma (MSKCC, 2020)

Lung Adenocarcinoma (MSKCC, 2021)

Lung Adenocarcinoma (MSKCC, Science 2015)

Lung Adenocarcinoma (NPJ Precision oncology, MSK 2021)

Lung Adenocarcinoma (OncoSG, Nat Genet 2020)

Lung Adenocarcinoma (TCGA, Firehose legacy)

Lung Adenocarcinoma (TSP, Nature 2008)

Non-small cell cancer (MSKCC, Cancer discov 2017)

Lung squamous cell carcinoma (CPTAC, Cell 2021)

Lung squamous cell carcinoma (TCGA, Firehose Legacy)

**Prostate Cancer**:

Prostate Cancer (DKFZ, Cancer Cell 2018)

Prostate Cancer (MSK, Cell Metab 2020)

Metastatic Prostate Adenocarcinoma (MCTP, Nature 2012)

Metastatic Prostate Adenocarcinoma (SU2C/PCF Dream Team, PNAS)

Metastatic castration-sensitive prostate cancer (MSK, Clin Cancer Res)

Neuroendocrine Prostate Cancer (Multi-institute, Nat Med 2016)

Prostate Adenocarcinoma (Broad/Cornell, Cell 2013)

Prostate Adenocarcinoma (Broad/Cornell, Nat Genet 2012)

Prostate Adenocarcinoma (CPC-GENE, Nature 2017)

Prostate Adenocarcinoma (Fred Hutchinson CRC, Nat Med 2016)

Prostate Adenocarcinoma (MSKCC, Cancer Cell 2010)

Prostate Adenocarcinoma (MSK, Eur Urol 2020)

Prostate Adenocarcinoma (MSKCC, PNAS 2014)

Prostate Adenocarcinoma (MSKCC/DFCI, Nat Genet 2018)

Prostate Adenocarcinoma (SMMU, Eur Urol 2017)

Prostate Adenocarcinoma (TCGA, Firehose Legacy)

Prostate Adenocarcinoma Organoids (MSKCC, Cell 2014)

Prostate Cancer (MSKCC, JCO Precis Oncol 2017)

The Metastatic Prostate Cancer Project (Provisional, November 2019)

**Skin Melanoma**:

Acral Melanoma (TGEN, Genome Res 2017)

Metastatic Melanoma (DFCI, Nat Medicine 2019)

Melanoma (Broad/Dana Farber, Nature 2012)

Melanoma (MSKCC, Clin Cancer Res 2021)

Melanoma (MSKCC, NEJM 2014)

Melanomas (TCGA, Cell 2015)

Metastatic Melanoma (DFCI, Science 2015)

Metastic Melanoma (MSKCC, JCO Precis Oncol 2017)

Skin cutaneous Melanoma (Broad, Cell 2012)

Skin cutaneous Melanoma (TCGA, Firehose Legacy)

Skin cutaneous Melanoma (Yale, Nat Genet 2012)

Skin cutaneous Melanoma (Broad, Cancer Discov 2014)

Desmoplastic Melanoma (Broad Institute, Nat Genet 2015)

**Uterine Cancer**:

Endometrial Cancer (MSK, 2018)

Endometrial Cancer (CPTAC, Cell 2020)

Uterine Corpus Endometrial Carcinoma (TCGA, Firehose Legacy)

Uterine Carcinosarcoma (John Hopkins, Nat Commun 2014)

Uterine Carcinosarcoma (YCGA, Firehose Legacy)

Uterine Clear Cell Carcinoma (NIH, Cancer 2017)
